# Pseudorandom sequence contention algorithm for IEEE 802.11ah based internet of things network

**DOI:** 10.1371/journal.pone.0237386

**Published:** 2020-08-13

**Authors:** Mohammed A. Raouf, Fazirulhisyam Hashim, Jiun Terng Liew, Kamal Ali Alezabi

**Affiliations:** 1 Department of Computer and Communication Systems Engineering, Faculty of Engineering, Universiti Putra Malaysia, Seri Kembangan, Malaysia; 2 Wireless and Photonics Network Research Centre (WiPNET), Universiti Putra Malaysia, Seri Kembangan, Malaysia; 3 Institute of Computer Science and Digital Innovation (ICSDI), UCSI University, Seri Kembangan, Malaysia; University of Electronic Science and Technology of China, CHINA

## Abstract

The IEEE 802.11ah standard relies on the conventional distributed coordination function (DCF) as a backoff selection method. The DCF is utilized in the contention-based period of the newly introduced medium access control (MAC) mechanism, namely restricted access window (RAW). Despite various advantages of RAW, DCF still utilizes the legacy binary exponential backoff (BEB) algorithm, which suffers from a crucial disadvantage of being prone to high probability of collisions with high number of contending stations. To mitigate this issue, this paper investigates the possibility of replacing the existing exponential sequence (i.e., as in BEB) with a better pseudorandom sequence of integers. In particular, a new backoff algorithm, namely Pseudorandom Sequence Contention Algorithm (PRSCA) is proposed to update the CW size and minimize the collision probability. In addition, the proposed PRSCA incorporates a different approach of CW freezing mechanism and backoff stage reset process. An analytical model is derived for the proposed PRSCA and presented through a discrete 2-D Markov chain model. Performance evaluation demonstrates the efficiency of the proposed PRSCA in reducing collision probability and improving saturation throughput, network throughput, and access delay performance.

## Introduction

With the emergence of Internet-of-Things (IoT) and Machine-to-machine (M2M), the number of wireless stations has increased explosively. Wireless network resources are to be shared by a large number of IoT-based stations, such as sensors, actuators, smartphones, and smart appliances [1]. These stations are associated to various fields, such as home automation, industrial and manufacturing automation, smart grid and metering, e-health, data analysis and management, security systems, intelligent transportation systems, public surveillance, etc. [2, 3]. Thus, for these various application scenarios, suitable wireless access technologies are required that provide a good quality of service (QoS) and reliable connection.

High data throughput is one of the major challenges for IoT network designers, given the heterogeneity and large quantity of stations. In the widely-implemented contention-based protocols, packet collisions are the main reason for data throughput degradation. In addition, channel access delay is considered as a major factor for many IoT use case scenarios such as smart automotive systems, smart healthcare systems, smart monitoring and tracking systems, in which these time-sensitive applications are necessarily in need with ultra-low delay wireless network services [4].

IEEE 802.11 is an efficient candidate to promise good QoS in various environments, whether indoor or outdoor, thereby the lately released standard of IEEE 802.11ah (or WiFi HaLow) is selected to be the technology that serves under IoT circumstances. Knowing that IEEE 802.11 standards are using conventional protocols and schemes in both data link and physical layers, IEEE task group ah (TGah) has introduced novel MAC protocols that can adaptively operate under various traffic patterns and user loads, also provide the necessary requirements of future IoT network scenarios.

To solve the bottleneck of contention-based protocol, Restricted Access Window (RAW) mechanism is introduced for the aim of reducing or mitigating collisions by dividing the large number of stations into smaller groups. Nonetheless, the RAW protocol is depending on the traditional distributed coordination function (DCF) and the enhanced distributed channel access (EDCA) [5]. Both channel access methods of DCF and EDCA utilize the algorithm of binary exponential backoff (BEB) despite the fact that many researchers have shown and proved that BEB has a major drawback of very high collision probability [6].

Fundamentally, the BEB algorithm for backoff selection is about doubling up the contention window (CW) size after every failure in the data packet transmission attempt. In the early backoff stages, if there are a large number of stations contending for the channel, this doubling process is crucial to reduce the collision probability. In the case of continuous collision, the station is forced to choose a larger CW size due to the rapid exponential increment until it reaches the maximum value of CW size. However, when a transmission successfully is accomplished, the CW size will be reset to the initial minimum value, which can lead to a high possibility of collisions. In light of this, BEB is vulnerable to high data packet collision. These disadvantages of the BEB algorithm prove that this traditional scheme may not be adequate for high-density wireless networks. Knowing that most of the wireless systems in future IoT scenarios are sensor-enabled systems in which there are constraints of power source usage such as sensors that operate on batteries. So, there are more difficulties in terms of the ability to sense the shared medium for a long duration continuously.

In this paper, we propose a backoff algorithm that employs a pseudorandom sequence (PRS) generated by a linear feedback shift register (LFSR), referred to as the pseudorandom sequence contention algorithm (PRSCA), to replace BEB algorithm and eventually to improve IEEE 802.11ah WLAN QoS performance. The proposed PRSCA algorithm aims to improve the QoS parameters of throughput and delay through a more developed solution, while still can be implemented easily. The PRS is used due to its better characteristics comparing to the exponential sequence of traditional BEB in terms of rate of growth, fast integers generation, and high quality of sequence that can adapt with various congestion scenarios. Thereafter, a discrete 2-D Markov model is developed with inspiration from the well-known model of Bianchi [7] and similar to that of Gopinath et al. [6].

The major contributions of this paper are outlined as follows: We propose the use of a pseudorandom sequence to be an alternative for BEB sequence; We propose an efficient backoff algorithm using the pseudorandom sequence to calculate the contention window size; We compute and analyse the collision probability and normalized saturation throughput of our proposed backoff algorithm with a 2-D Markov model and compare the results with BEB algorithm; We investigate the QoS performance notably throughput and delay of the proposed algorithm via MATLAB.

The remainder of the paper is organized as follows. Section 1 provides an overview of the IEEE 802.11ah use cases and MAC features. Section 2 summarizes existing work. Section 3 presents the proposed backoff algorithm and its characteristics, also analytical expressions to compute collision probability and saturation throughput. Section 4 describes the simulation setup details, also analysis and discusses the results. Finally, in the last section, conclusions are given in Section 5.

## 1 Background

This section presents an overview of IEEE 802.11ah, notably its use case scenarios, IEEE 802.11ah MAC features, Restricted Access Window (RAW), and Binary Exponential Backoff (BEB) algorithm.

### 1.1 IEEE 802.11ah use case scenarios

IEEE 802.11ah (or WiFi HaLow) is introduced to meet and satisfy the requirement of future M2M and IoT communication. Its novel and innovative features enable WiFi HaLow to support a large number of stations in a dense network environment. In particular, various new features have been introduced in WiFi HaLow physical and MAC layers (to be discussed in the next section) that make it appropriate for a highly dense environment with long-extent wireless networks of stations such as IoT enabled applications [8, 9, 10]. One of the adopted scenarios by TGah is the sensor networks because of the high penetration ability of WiFi HaLow through obstacles and high-density areas. Additionally, the deployment of networks can have more stations in only one hop model. Therefore, low power sub-GHz vendors for smart WSNs situations can comply with an identical communication technology that is able to provide connectivity to applications with high demand. Moreover, several industrial applications are able to get benefits from the adaptability of WiFi HaLow technology.

**Fig 1:**
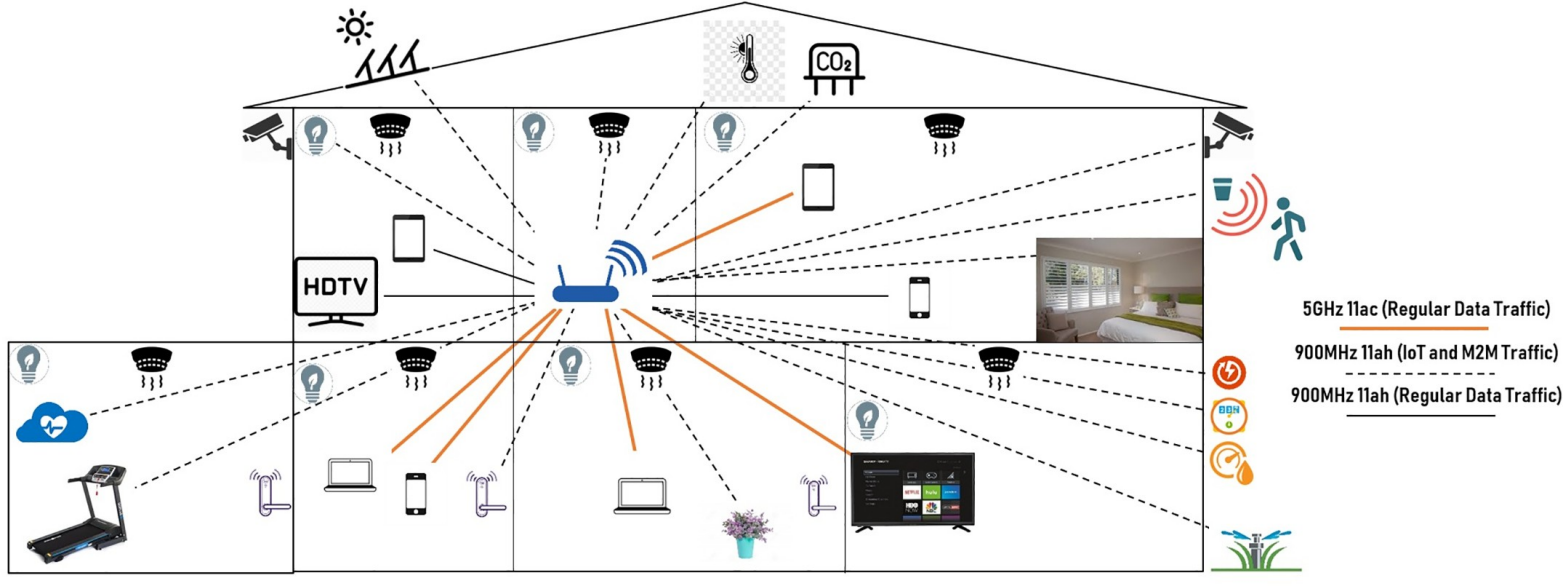
Tri-Band Wi-Fi AP using IEEE 802.11ah for home automation.

Besides, many types of small-scale objects and appliances can be deployed with radio sensors, so that indoor/outdoor home automation becomes possible as advertised by the WLAN industries [11, 12]. Residents of a house can easily control and receive data from small devices embedded with sensors such as temperature sensor, humidity sensor, CO2 sensor, water meter sensor, power meter sensor, gas meter sensor, smart lighting on/off sensors, win-dow shelter sensor, smoke detector sensors, smart locking system for entrance doors, security camera and motion sensors, smart sprinkler sensor, moisture sensors, heart rate monitoring sensor, smart fitness tracker, solar panel sensor, etc. Similarly, see Fig 1.

### 1.2 IEEE 802.11ah MAC features

Given the lack of dedicated link in wireless communication, the MAC protocol is required to coordinate the channel access shared by the wireless stations. Major enhancements and changes are made in IEEE 802.11ah to address the requirements of future M2M and IoT are in the MAC layer. The main challenge for the TGah is the task of supporting an overwhelming amount of stations in transmitting multiple short data packets in a timely manner while under power constraints. The MAC layer of IEEE 802.11ah suitably designed by TGah to break many formal constraints of protocol overhead, ideal listening, overhearing, and power efficiency.

#### 1.2.1 IEEE 802.11 collision reduction mechanisms

In this sub-section, we discuss some common MAC layer protocols and solutions designed by 802.11 task groups. When multiple stations shared the wireless channel for sending and receiving of data frames, coordination is needed through a multiple access protocol. Accordingly, 802.11 designers selected a random-access protocol called carrier sense multiple access with collisions avoidance (CSMA/CA). CSMA/CA means that prior to the process of transmitting a frame, every station must sense the shared medium, and abstain from data transmitting when the medium is sensed to be reserved by other stations or busy [13]. The process is handled by using a schematic acknowledgement and retransmission techniques, and Automatic Repeat Request (ARQ) protocol. The moment that a station starts to send a frame, the station sends the whole frame, and there are no regressions. Therefore, whenever long frames are transmitted and collisions happen frequently, the multiple access protocol will certainly degrade in performance.

**Fig 2:**
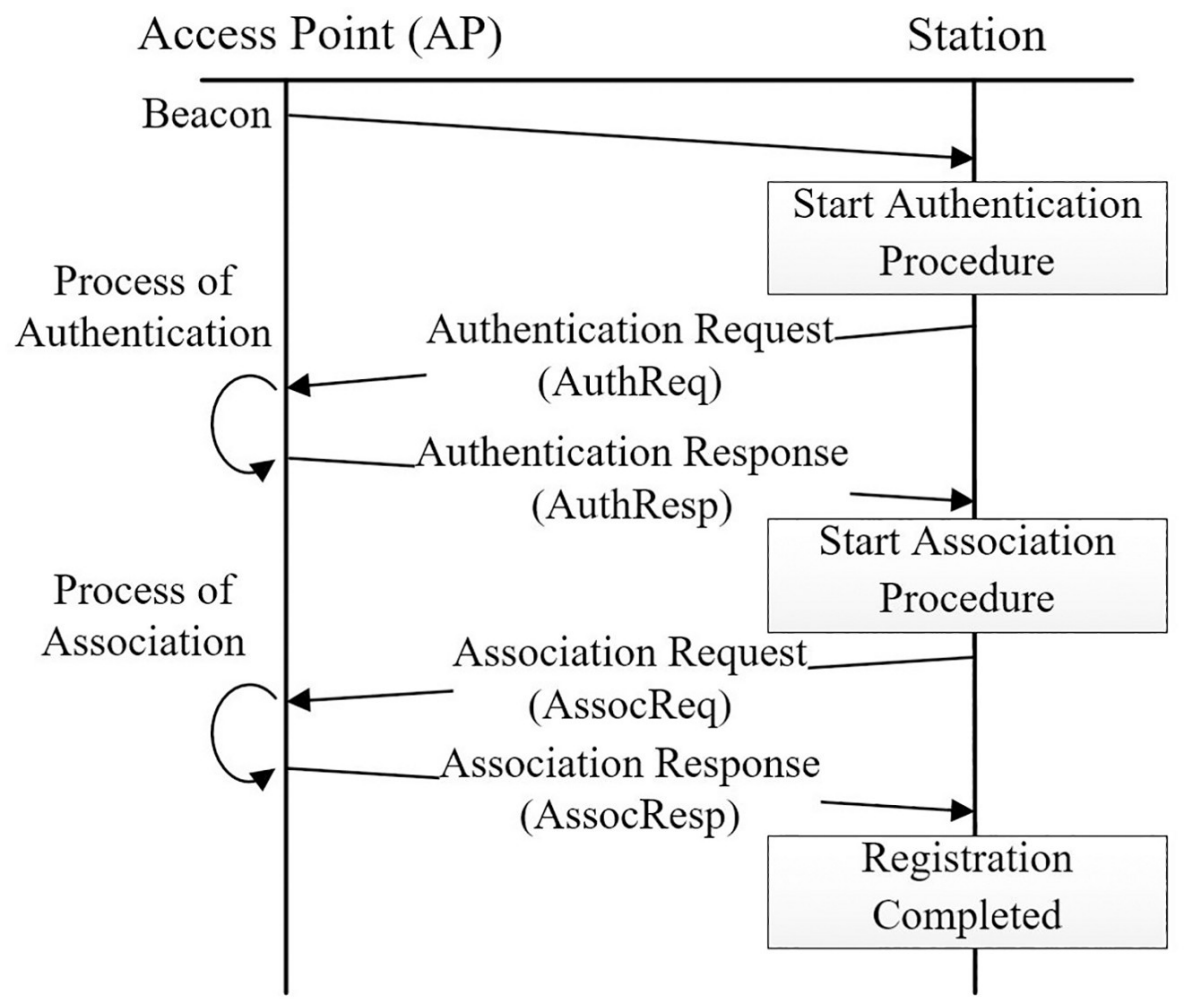
IEEE 802.11ah registration procedure [14].

Apart from packet transmission, CSMA/CA is also used in the wireless station registration process with the access point (AP) [14]. The registration process is performed by the station prior to any data packet transmission. Fig 2 shows the registration process implemented in IEEE 802.11ah protocol. It is a four-way handshake mechanism, which includes authentication request (AuthReq), authentication response (AuthResp), association request (AssocReq, and lastly, an association response (AssocResp). Both AuthReq and AssocReq are transmitted by the wireless station, while AuthResp and AssocResp are sent by the AP. Similar to packet transmission, the transmission of request/response messages are using CSMA/CA for random access protocol. Therefore, the performance of CSMA/CA has a huge impact on the overall network performance.

**Fig 3:**
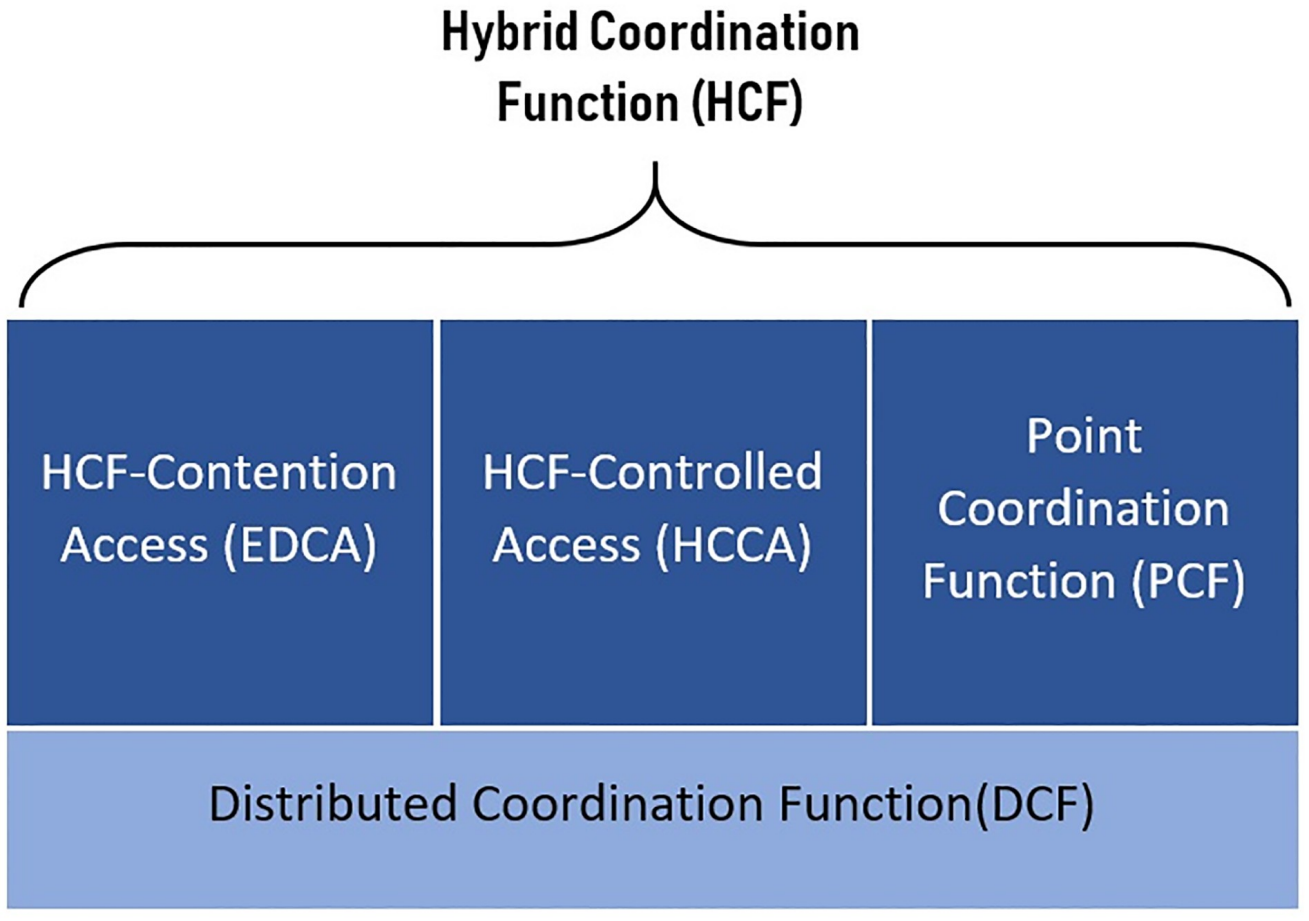
IEEE 802.11 MAC architecture.

Based on the coordination mechanism, the MAC protocol can be divided into different groups: contention-based, contention-free, and hybrid-based protocol. In Fig 3, an architecture representation of MAC protocols of the coordination functions is shown.

The fundamental method of channel access with contention-based is provided by the distributed coordination function (DCF) [15]. DCF channel access method manages asynchronous network traffic with best effort, where all stations attain similar opportunities to access the channel. On the other hand, another method is optional support of channel access with contention-free, provided by the point coordination function (PCF) alongside polling control-based protocol [16]. In PCF, real-time requirements can be met and settled by the AP for use cases of voice communications and live streaming of videos. Then both methods of contention-free and contention-based are combined by the hybrid coordination function (HCF), which is a replacement of PCF. HCF is a composition of the hybrid-controlled channel access (HCCA) function controlling the contention-free channels and the enhanced distributed channel access (EDCA) function controlling the contention-based channels. Both functions have the target of offering quality of service (QoS) requirements [17], where transmissions with priorities are supported by defining categories of network traffics [18].

#### 1.2.2 Enhanced collision reduction mechanisms in IEEE 802.11ah

**Fig 4:**
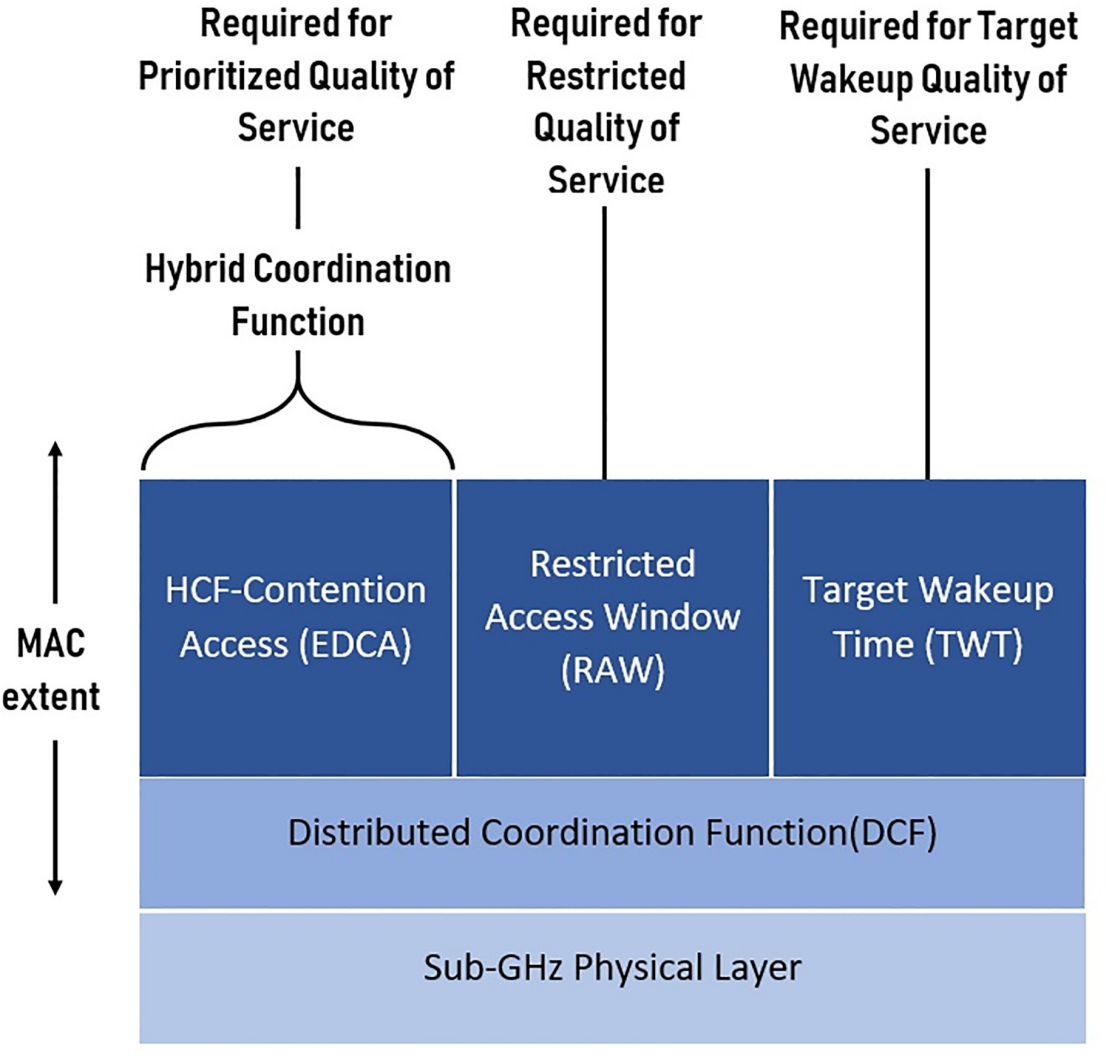
IEEE 802.11ah MAC architecture.

As a novel IEEE 802.11 standard, the TGah defined this amendment in terms of quality in supporting a large number of stations with transmissions of small data packets. Former MAC protocols of 802.11 standards act as a dependable base for the 802.11ah standard, but there are some limitations of using these protocols for future IoT network environments with a high density of stations. Therefore, within the development process of this new 802.11 amendment, the major effort was to reduce collision probability and frame overhead, also adding energy efficiency improvements. Fig 4 illustrates the MAC architecture of 802.11ah. Two protocols, namely restricted access window (RAW) and target wake time (TWT) are defined for enabling IoT technology using the procedure of both DCF and EDCA access methods in WLAN channels [19, 20]. As explained in the earlier section, outdoor network use cases have stations in tremendous amount, where many hidden stations in the area of coverage cause data packet collisions during their uplink transmission attempts. In fact, channel efficiency can be improved by reducing potential data packet collisions through restricting access to the shared medium for a small group of stations over a pre-defined time period. Consequently, the uplink transmission attempts of the stations are distributed.

Therefore, stations are classified and defined into three various types by 802.11ah standard; each one of these types has different durations and processing steps to access the same shared medium. They are indicated as traffic indication map (TIM), non-TIM stations, and non-scheduled station. Fundamentally, the RAW and TWT concepts are to distribute the attempts of uplink accesses over a lengthy time period.

In the operation of RAW, a station is required to wait for its turn according to a pre-defined time slot schedule of transmissions and remains idle outside its assigned time slot. Likewise, in TWT, station activity is to be controlled and managed within the basic service set (BSS), where stations are scheduled to initiate their transmission at various time periods and remain in sleep mode outside the active time for power-saving. So, these protocols are defined in order to reduce both of contention among multiple stations and the operational power.

### 1.3 Restricted Access Window (RAW)

As mentioned earlier, RAW is introduced to mitigate high channel contention and collisions, and hidden node issues in dense WLAN. Basically, the process is outlined as an access point (AP) broadcast information element (IE) over short frames of beacons, where many RAW sets are allocated and initiated. Each RAW set is divided and sectioned into even slots of time in which each slot of time can be assigned to one station or a group of stations. A station follows channel access methods of DCF/EDCA within the assigned RAW duration. The protocol of RAW prevents data packet transmissions from overlapping among the number of contending stations, and the problem of hidden node is alleviated by restricting the duration that a contending station is allowed to initiate transmission. This is why RAW requires to use group-based contention strategies [21].

**Fig 5:**
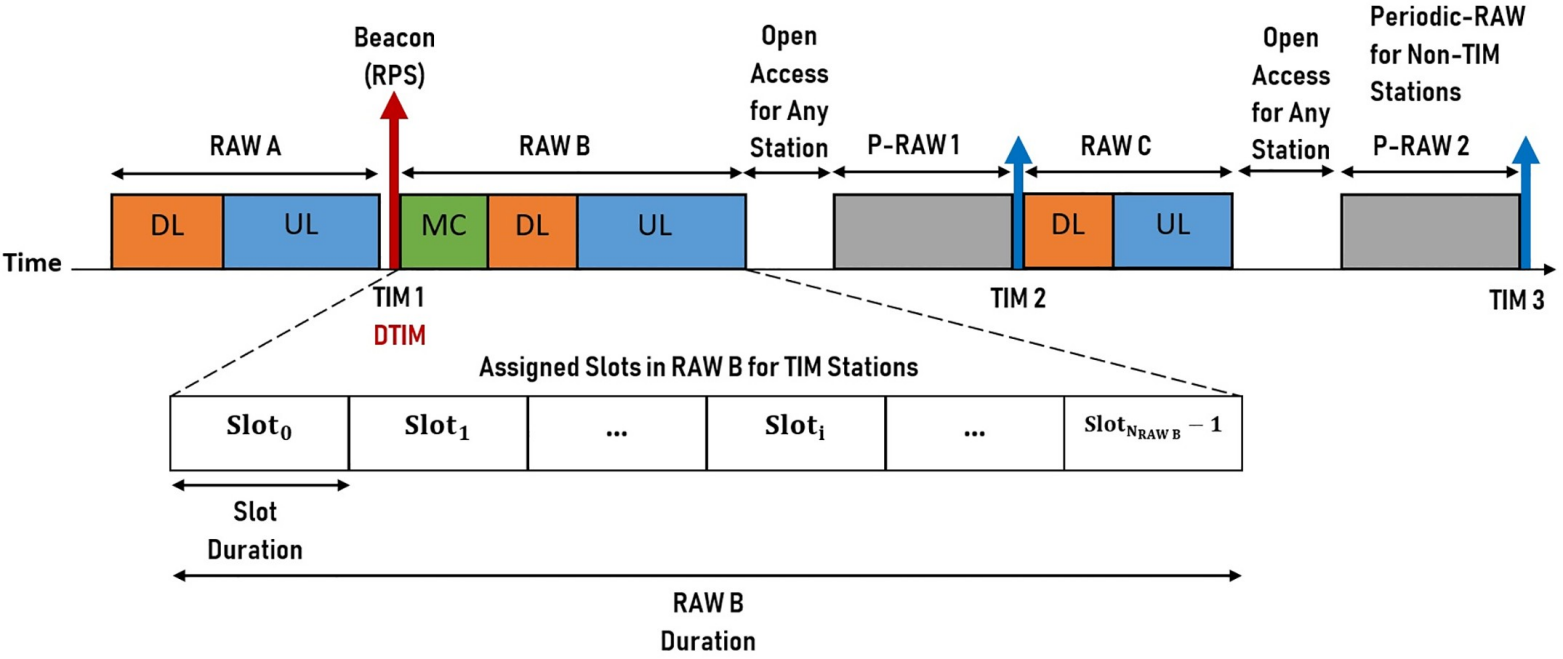
The distribution of RAW structure between signalling.

The RAW structure is illustrated in Fig 5. The depicted figure shows the working principle of RAW, where the airtime is divided into multiple TIM intervals [22]. Every TIM interval is allocated with a single RAW group for TIM stations and a periodic-RAW (P-RAW) for non-TIM stations. There is a contention-free channel access duration opens for any stations, especially those with high priority traffics [9]. RAW groups can be slotted into sub-slots, which are typically assigned to a single TIM station or more TIM stations. The highest quantity of RAW slots is set to be 64. At the starting time of the assigned slot of RAW, station within the RAW group is allowed to contend for the channel.

An allocated station is required to implement the contention-based method to access the medium. There is essential RAW information that will be broadcasted, which consists of the number of RAW slots, the duration of RAW slots, and the time when the RAW group starts. This information is broadcast in beacons by the AP using the IE of RAW parameter set (RPS) [23]. At periodic intervals, every station in the WLAN listens to receive the information of RAW scheduling from each broadcast frame of beacon referred to as Target Beacon Transmission Time (TBTT). When the allocated RAW duration expires, every station in the group has to switch into the dozing state (sleep mode) and remain idle until the beginning of their next assigned RAW. Particularly using this mechanism of RAW can alleviate collisions between hidden nodes [24].

Within a RAW duration, it is forbidden for any station to access the medium outside its own assigned time slot. Considering uplink (UL) transmissions, station attempts for channel access stations are required to select a slot randomly in the window that is allocated. While for downlink (DL) transmissions, slots are assigned for data packet transmission from the AP to the station [25]. RAW is formed and organized in every TIM by a single segment of UL, and a single segment of DL, also directly after each beacon of DTIM a single segment of multicast (MC) is located where transmitting and receiving of data packets are allowed together [9].

### 1.4 Binary Exponential Backoff (BEB) algorithm

802.11 WLANs adopts the BEB scheme. At each packet transmission, the station uniformly chooses the backoff time in the range (0,*CW*_*min*_ − 1), where *CW*_*min*_ is the minimum contention window. The identical range of CW promises the contending stations with equal transmission probability. After each unsuccessful transmission or collision, the CW will be doubled up until successful transmission, or to the maximum contention window, *CW*_*max*_. For every attempt of data packet transmission, a new CW is calculated as in Eq 1:

$$\begin{array}{c} CW=min[2\times CW,CW_{max}] \end{array}$$ (1)

The maximum backoff stages allowed is referred to as *m*. Note that *m* has a limit of 7 stages in the mechanism of basic access, and 4 stages in the handshake mechanism of RTS/CTS used in both DCF/EDCA [26]. In the BEB algorithm, after a successful data packet transmission, the station resets its CW size to *CW*_*min*_. Nevertheless, this resetting mechanism results in significant changes in the CW size and consequently leads to network performance degradation. When there is a heavy traffic load in the shared channel, every new data packet transmission begins with *CW*_*min*_ value [27]. Smaller CW size can increase the chance of collision due to the higher likelihood of overlapping CW size. Subsequently, when the amount of collisions reaches *m*, the required data packet for transmission must be discarded.

During the channel contention, the station senses the medium continuously, and whenever the medium is idle, the station decreases the BOC by one. When the BOC arrives at zero, the station can access the channel and begins to transmit the required data packet.

Fundamentally BEB backoff mechanism designed in a way that, upon any data packet collision, the value of CW is calculated as *CW_i_* = 2^*i*^ × *CW_min_*. While upon successful transmission the CW size is determined as *W*_*i*_ = *CW*_*min*_. There are some drawbacks in BEB that are worth to be mentioned. Firstly, the rapid growth in the value of CW can cause the average delay to increase exponentially [6]. Besides, BEB deals with any noise corruption as a frame collision, so this increases its vulnerability to more frame collision. Fairness between stations is considered as another issue with BEB. Several researches on the BEB algorithm have been conducted in terms of the network performance since the traffic load raises, and the number of stations vastly increases. In the following section, several related works are reviewed and highlighted in a similar concern. Existing works that concentrate on the stability and instability of this exponential doubling mechanism under various network conditions will be discussed.

## 2 Related work

This section presents some recent works that mitigated the drawbacks of the BEB algorithm in IEEE 802.11 standards, including IEEE 802.11ah [28–36]. As mentioned earlier, the IEEE 802.11ah MAC protocol of RAW reduces collisions in highly congested network scenarios by using EDCA and DCF access schemes to manage transmission attempts. In EDCA, two backoff-counter are used; the first backoff counter is used outside of the RAW slot, and the second one is used inside the slot [28]. Multiple stations can be assigned within a RAW slot, and they need to contend within their associated slot to have access to the shared channel. In order to solve contention among the stations, BEB is used as the backoff algorithm; however, this traditional BEB encounters a high probability of collisions.

In [34], the authors highlighted that the algorithm of BEB is not efficient enough to sustain the upcoming wireless LAN with a heavy load of network traffic, mainly due to its resetting mechanism of CW size to the minimum value after every data packet successfully transmitted. This inefficiency leads to degradation in the overall performance of the network because of higher collisions. The authors subsequently proposed a channel observation-based scaled backoff (COSB) algorithm to facilitate WLANs with higher efficiencies in terms of throughput and delay. This COSB mechanism is utilized to variate the scale of the window size in an adaptive manner based on the dynamic channel condition. However, the implementation of this algorithm still makes use of the BEB sequence, which can cause a drastic increase of CW with every collision experienced.

A self-adaptive backoff algorithm (SABA) was proposed in [29] to update the CW size based on instantaneous network status, in which any variation happens in the size of network regarding active stations throughout the running time, the CW value must be adjusted to the optimum factor. The simulated results showed that SABA outperforms BEB in terms of improving the throughput and delay performance by reducing the collisions in the network. In [30], the authors proposed a centralized random backoff (CRB) algorithm as a multiple access protocol used for a WLAN of collision-free. With the help of acknowledgement frame, the station is allocated to a unique backoff state by the AP. The results indicated that by reducing collisions, the throughput performance is significantly improved. In the meantime, without dynamic adjustment of parameters, all stations in larger numbers are allowed to run within a state of collision-free.

Studies of deep learning are well documented, and its potential implementation in the WLAN MAC layer has been investigated. Authors of [35] have proposed an intelligent paradigm of reinforcement learning (RL) for resource allocation of the MAC layer. Within this paradigm, a MAC scheme of channel observation was utilized so the performance can be optimized. Additionally, a mechanism of resource allocation was proposed. While the results shown are very promising, the feasibility of the scheme remains uncertain due to the low processing capability of wireless stations subjected to energy constraints.

 [36] also emphasised that the current BEB algorithm fails to accomplish higher efficiencies in both scattered and congested wireless networks. The authors have highlighted the problem is due to the exponential increase of the window size that leads to minimizing throughput in use cases of high-density networks and causing unwanted delays in use cases of sparse networks. Taking into account both use cases, the CW size needs to be adaptively determined. Therefore, a mechanism of cognitive backoff (CB) was proposed. NS3 is used alongside a detailed performance analysis, concentrating on achieving high throughput and low delay compared to the BEB algorithm.

In IEEE 802.11ah WLANs, the majority of stations are set to remain in sleeping mode outside their allocated RAW slot. However, at the beginning of each RAW slot, the network suffers from a high probability of collisions due to the possibility of simultaneous channel access by the assigned station. Therefore, this kind of threat needs to be mitigated. The authors in [32] proposed a combination mechanism of RAW and adaptive scheme used to calculate an optimal value of the CW size at the beginning of the beacon interval, then slowly halving it when the transmission is successful. The performance of the proposed scheme has been evaluated using the OPNET simulator. From the simulation results, it can be observed that the proposed scheme significantly improved the network performance compared to the legacy MAC protocol of DCF with respect to media access delay and retransmission attempts.

A self-adapting MAC protocol (SaMAC) is proposed in [33], which utilized shifted contention window mechanism that enhanced constrained countdown freezing. A multi-dimensional Markov chain modeling is proposed to model the SaMAC protocol, and through several network scenarios, the accuracy of the proposed model has been investigated. The performance characteristics of the proposed protocol have been evaluated using the MATLAB software. It is shown from the simulation and analysis that SaMAC protocol offered better throughput, fairness, and jitter than the legacy DCF protocol while keeping simple implementation.

Lastly, to help facilitate the Quality of Service (QoS) in wireless networks, a MAC protocol called Enhanced Distributed Channel Access(EDCA) was introduced. However, the core backoff algorithm used in EDCA is still the BEB algorithm. Hence, even though EDCA allows the prioritisation of channel access, it is still susceptible to the problem of BEB, such as the CW resetting issue and inefficiency of the BEB sequence. Authors of [37] proposed a mechanism of machine-learning-enabled EDCA (MEDCA) where the CW size is adjusted adaptively according to the WLAN density. They have presented their results considering QoS parameters and showed that their proposed method performed better when compared to conventional EDCA mechanism.

## 3 Proposed PRSCA backoff algorithm

### 3.1 Problem formulation

**Fig 6:**
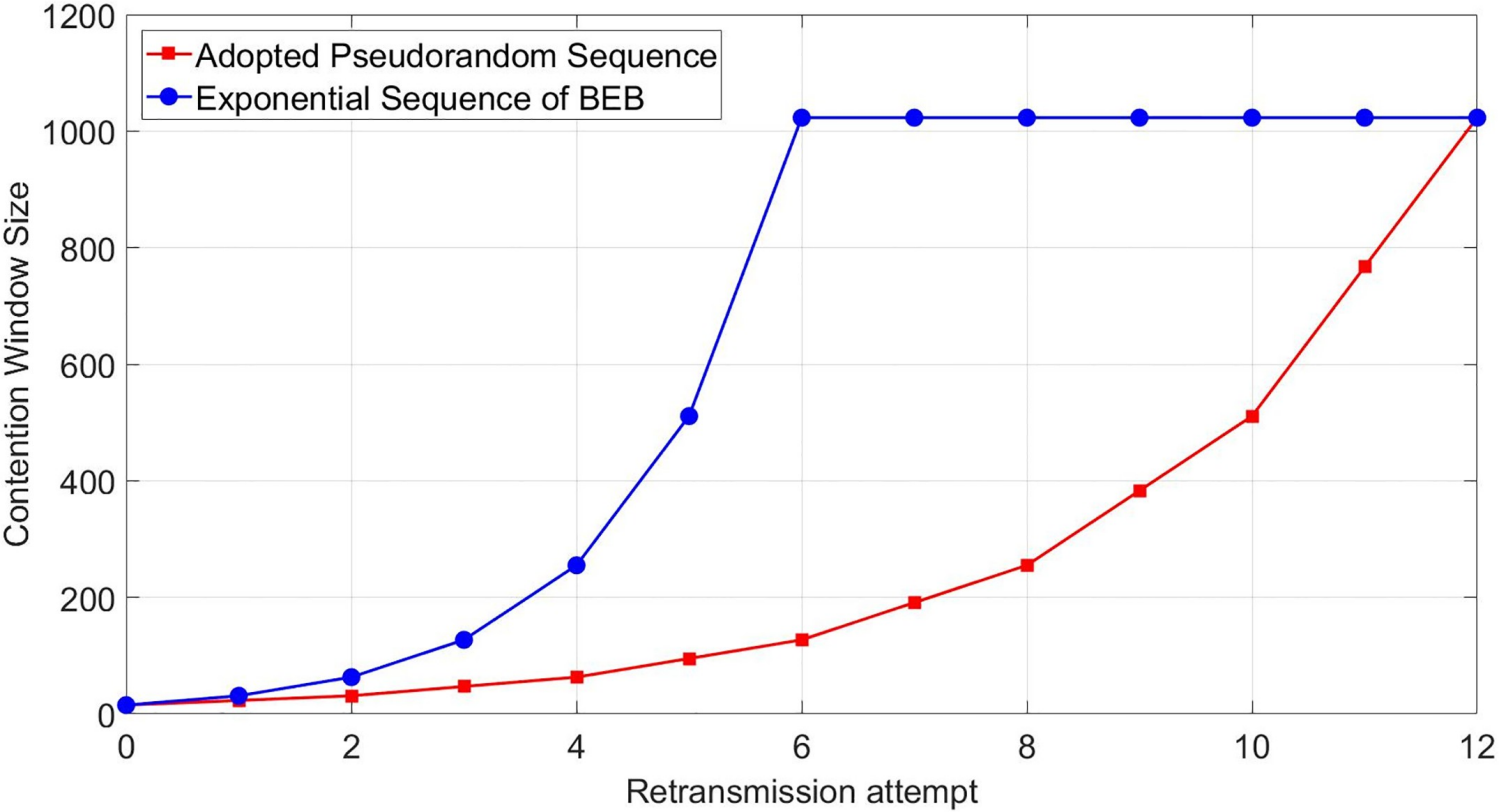
CW size against retransmission attempt.

As aforementioned, the CW size increases exponentially with any data packet collisions in the BEB algorithm. This increase in the CW size phenomenon leads to an unnecessary idle duration in the channel, which downgrades the QoS of networks and may not be adequate for ultra-dense IoT network environments. Moreover, the energy constraints of sensor nodes don’t allow a station for incessantly sensing the channel for a long duration. Fig 6 shows the changes in CW size according to the retransmission attempt using different algorithms. The drastic change of CW in the BEB sequence is the main culprit of prolonging the period spent during the decrement of backoff counter and channel sensing activity.

A specification of CW is that its value is predetermined to the minimum size referred to as CWmin in the initial state. Then, for each transmission failure, the CW size is doubled and growing continuously until the CW size reaches the maximum value of *CW*_*max*_, where the size is 1023. When the channel is unused or empty, the backoff counter (BOC) starts to decrement by one. While for the case of a transmission of the data packet that is successfully completed, the size of CW is reset to the initial window size of *CW*_*min*_. Consequently, this resetting is highly considered the main cause of data collisions in the BEB algorithm. As shown in the below figure, the station goes back to the initial stage and chooses the minimum backoff window size *CW*_*min*_ after the transmission has been performed successfully.

The reset of the backoff stage and *CW*_*min*_ can render the variation of the CW of the contending to be very low, hence, causing more simultaneous channel access upon depletion of the backoff counter. In light of these issues, an efficient backoff method is proposed, which uses a suitable pseudorandom sequence generated by the LFSR rather than the traditional BEB sequence. The proposed PRSCA backoff mechanism incorporates two main components, namely, a Linear Feedback Shift Register (LFSR) sequence to replace the conventional BEB, and backoff stage resetting.

### 3.2 Linear feedback shift register sequence

Our major motivation to choose this sequence is that we are relying on the growth speed of the sequence integers, which makes an efficient variation of CW values in the subsequent backoff stages. In addition, as the LFSR number increases, the sequence is suitable for either case of a small or large number of contending stations. Worth to mention that there are numerous types of sequences, some of them may not be suitable for this kind of usage because of their unwanted integer increments, while other sequences have limited impacts on the process of network performance improvement.

The LFSR is a sequence of scrambled linear pseudorandom integers, as defined in Eq 2. The LFSR integers are 1, 2, 3, 5, 7, 11, 15, 23, 31, 47, 63, 95, 127, 191, 255, 383, 511, 767, 1023, 1535, 2047… as stated in OEIS A052955 [38], where its closed form solution can be defined as follows [39]:

$$\begin{array}{c} PRS_{n}=n-\left\lfloor \frac{n}{2}\right\rfloor +\sum_{i=1}^{\left\lfloor \frac{n}{2}\right\rfloor }PRS_{n-2i} \end{array}$$ (2)

where *PRS*_1_ = 1 and *PRS*_2_ = 2. With simpler calculation *PRS*_*n*_ = 2 × *PRS*_*n*−2_ + 1, the pseudorandom integers can be determined by the recurrence correlation of the integers. The LFSR generates periodic sequence and can be created using Fibonacci model of sequences [40, 41]. Pseudorandom is a maximum length sequence generated by an LFSR and it has a characteristic of a primitive polynomial. Besides previous recurrence, particular integers of 2 and 3 are consecutively and repeatedly created by using this expression:

$$\begin{array}{c} C_{n}=\frac{5-{\left(-1\right)}^{n}}{2} \end{array}$$ (3)

Likewise, the exponential growth is determined as root of double integers created by using below expression:

$$\begin{array}{c} I_{n}=\frac{2\times n-1+{\left(-1\right)}^{n}}{4} \end{array}$$ (4)

The new CW for the proposed algorithm PRSCA can be calculated using this formulation:

$$\begin{array}{c} W_{i}=2^{I_{i}}\times C_{i}\times CW_{min} \end{array}$$ (5)

According to Eq 5, the calculation of CW by PRSCA for *i* numbers of backoff stage is formulated to be determined by the growth of multiplicative exponential increase of $2^{I_{i}}$ and the multiplicative of LFSR consecutive numbers of *C*_*i*_. In LFSR, the increment of generated integers is lesser compared to the exponential increment of BEB.

### 3.3 Backoff stage resetting process

In the proposed backoff resetting process, there is a revision of the procedure apart from the updated CW calculation mechanism. In conventional BEB, the backoff stage will reset to one, followed by an update of CW to *CW*_*min*_ for the contending station, which has successfully transmitted the packet. However, in the proposed backoff resetting scheme, the backoff stage of the successfully contending station will only reset to the backoff stage prior to the successful contention. This difference is especially apparent for the network with a high number of contending stations because the higher the number of stations, the higher the number of collisions. Hence, the higher the backoff stage for each station until successful contention.

**Fig 7:**
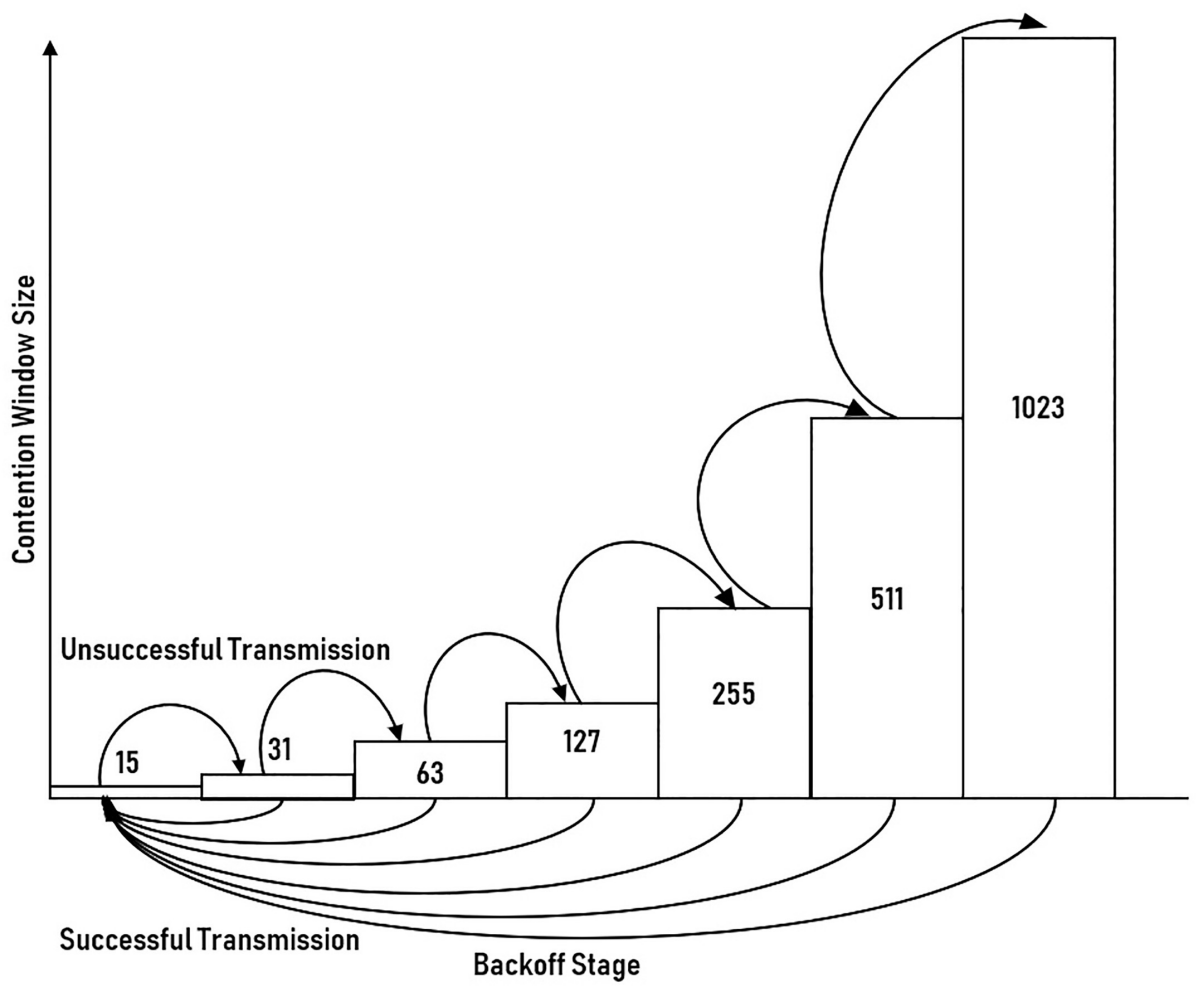
Backoff process of conventional BEB.

**Fig 8:**
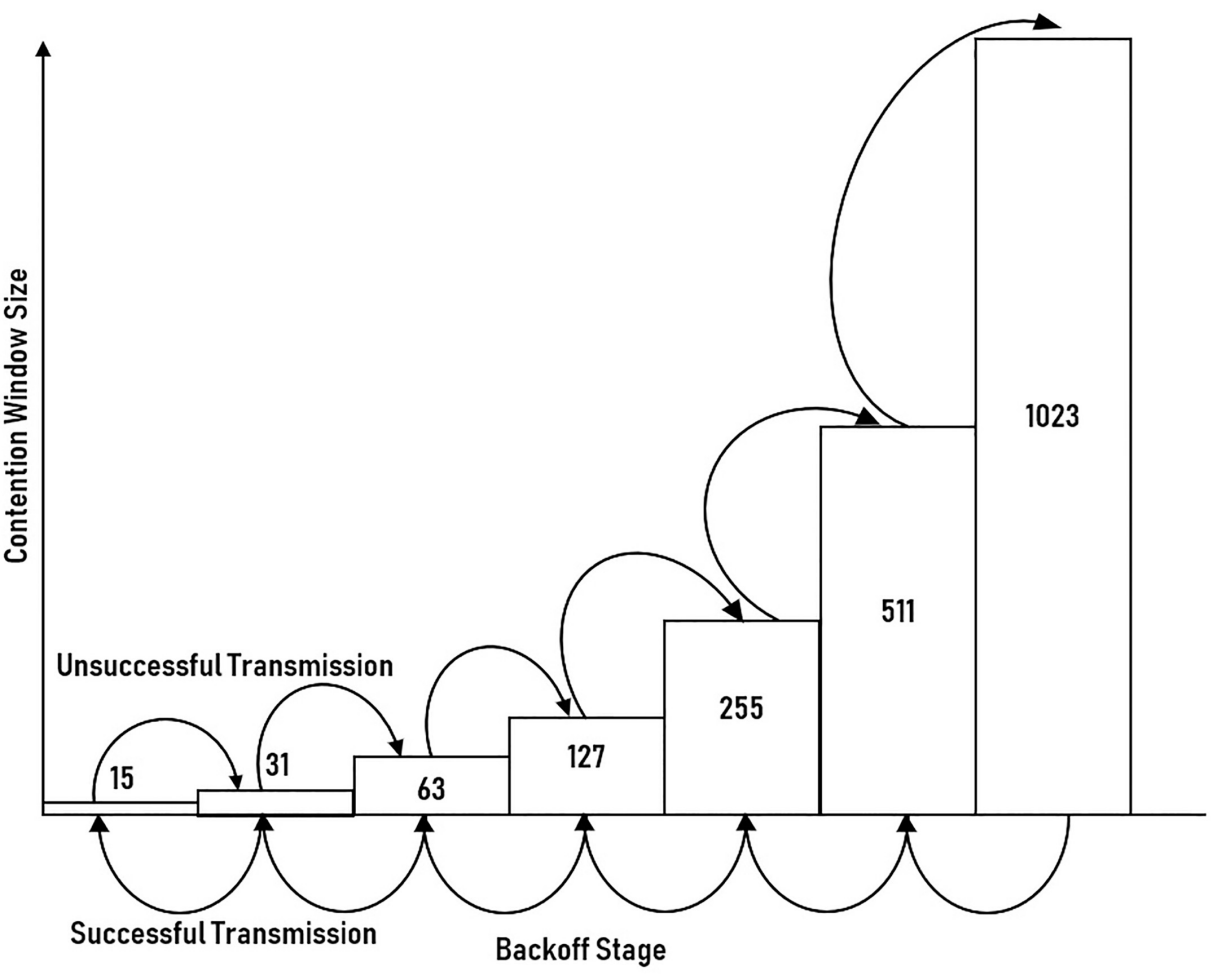
The backoff stage resetting process used in PRSCA.

In the network with a high number of contending stations, the high collision probability is expected, especially during the early backoff stages. This phenomenon is due to the fewer variation of CW that can be selected by the stations, hence, increase the chance of collision. To reduce the collision and expand the range of CW, the CW increases with the backoff stages. Therefore, the backoff stage during successful contention actually promises a suitable range of CW for contending stations, thereby signifies the instantaneous network condition. However, with the conventional BEB, the backoff stage will reset to the initial backoff stage, losing the network condition information in the process, as highlighted in Fig 7. In the proposed scheme, instead of resetting to the initial stage, the backoff stage will just reset to the backoff stage prior to successful contention, as shown in Fig 8.

**Fig 9:**
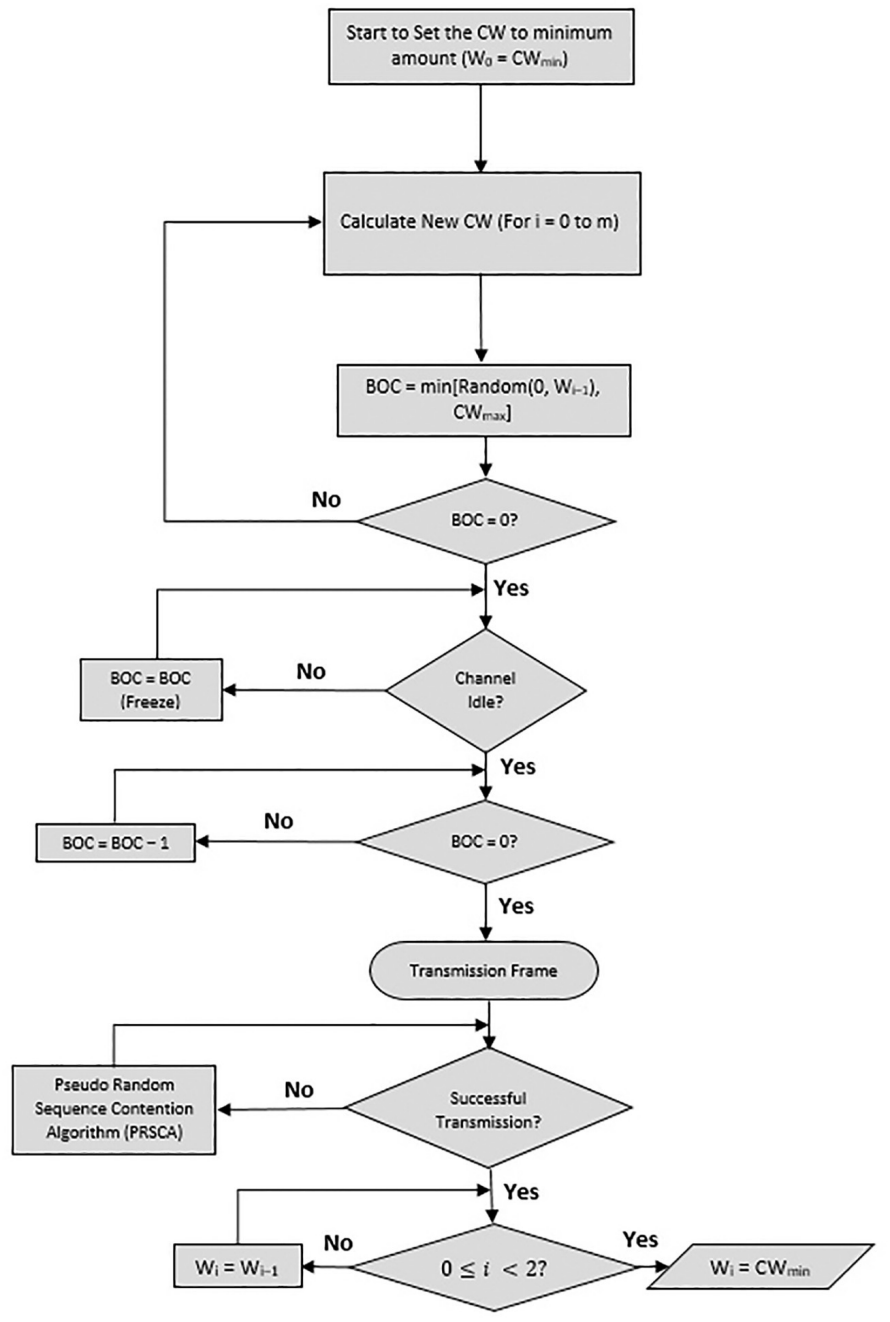
The proposed PRSCA backoff algorithm.

Following every transmission, i.e., either unsuccessful or successful, the size of CW will be updated by the proposed algorithm without relying on any former information. As illustrated by the flowchart in Fig 9, the proposed algorithm utilizes the LFSR rather than the exponential increment in the BEB algorithm. Next, we present the computation of CW size using the PRSCA, where substitutes PRS numbers. In PRSCA, the calculation of the window size will depend on multiplication to compute the new size of CW. In terms of hardware implementation, LFSR is very beneficial in applications that need a particularly fast PRS generation.

### 3.4 Analytical modeling

In this section, we present an analytical model for the proposed PRSCA algorithm. We assumed a fixed amount of *n* contending stations; every station has a data packet ready to be transmitted with a nonempty transmission queue in an ideal circumstance of the channel and no hidden terminals. We investigated the behaviour of the station by modeling it is using a 2-D discrete-time Markov chain. The stationary distribution probability is obtained wherein a randomly selected time slot a station sends a data packet using the DCF access method in a saturated condition.

**Fig 10:**
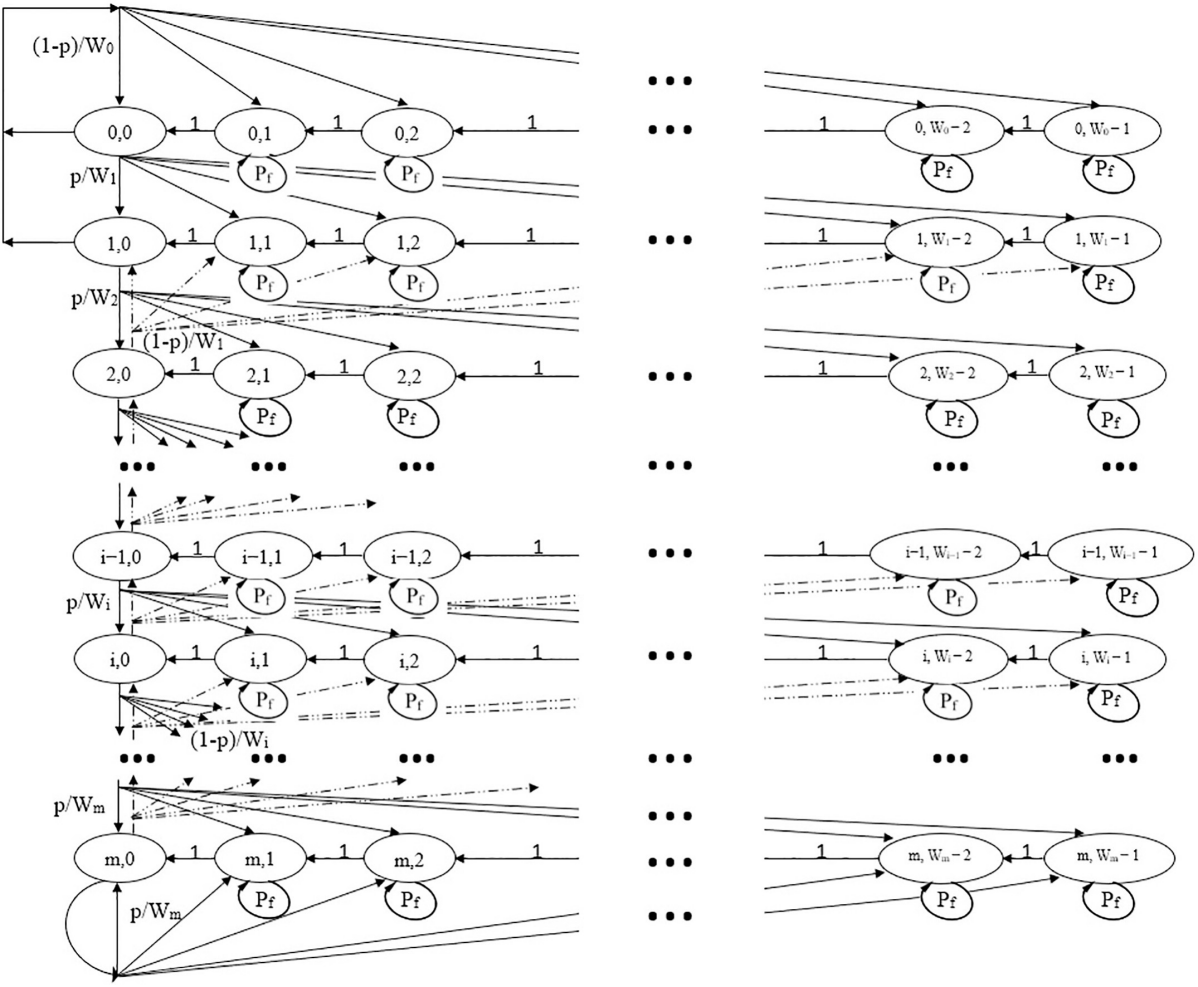
A 2-D Markov chain model of the proposed PRSCA backoff algorithm.

Fig 10 [6] illustrates the 2-D discrete-time Markov chain model. There are a finite number of transition states in certain time slot duration, and the moving probability of each state to the subsequent state relies on the prior state event. The potential transition from a state to another depends on two random processes. The first one is the backoff counter (BOC) identified as *b*(*t*) from 0 to CW−1, where the contention window size chosen within each stage. The backoff stage (BOS) is identified as *s*(*t*) from 0 to the largest number of stages denoted by *m*. Every state for any station is defined by *S*_*i*,*k*_, where the BOC is represented by *k*, and the BOS is represented by *i*.

In the following subsections, we discussed the derivation process of packet transmission probability, collision probability, and saturated throughput.

#### 3.4.1 Packet transmission probability

Possible backoff transitions are constructed as the following. If the shared medium is idle at any state defined by BOS (*i*) and BOC (*k* + 1), the BOC decrements by a value of 1 and changes to the subsequent state with a probability of 1. The BOC freezes if the station senses a busy shared medium at any state defined by *S*_*i*,*k*_. At the same state, with the probability of *P*_*f*_, the station BOC freezes, except for state *S*_*i*,0_. After each successful transmission, the station with its packet return back to first state *S*_0,*k*_, and the BOC is initialized to *CW*_*min*_ with the probability of 1 − *p*, and this happens in the first two stages. After a successful transmission the station selects its BOC value uniformly from the third BOS where *i* is equal to 2 with a probability of 1 − *p*, same as chosen in the prior BOS (*i* − 1, *k*); After an unsuccessful transmission at stage *i* − 1, with the probability of *p* the station chooses its BOC value from 0 to *W*_*i*−1_ within the subsequent stage of *i*; At all stages if the transmission is not successful, the station arrives at the final BOS and remains with the probability of *p* until the transmission of the data packet is successful. The two-dimensional method of both random processes of *s*(*t*) and *b*(*t*) is modelled with a 2-D discrete-time Markov chain model portrayed in Fig 10 [6]. The transition probabilities of this Markov chain are summarized herein:

$$\{\begin{array}{ll} P(i,k)|(i,k+1)=1, & fork\in (0,W_{i}-2),i\in (0,m)\\ P(i,k)|(i,k)=P_{f}, & fork\in (0,W_{i}-1),i\in (0,m)\\ P(0,k)|(i,0)=\frac{1-p}{W_{0}}, & fork\in (0,W_{i}-1),i\in (0,1)\\ P(i-1,k)|(i,0)=\frac{1-p}{W_{i}}, & fork\in (0,W_{i}-1),i\in (2,m)\\ P(i,k)|(i-1,0)=\frac{p}{W_{i}}, & fork\in (0,W_{i}-1),i\in (1,m)\\ P(m,k)|(m,0)=\frac{p}{W_{m}}, & fork\in (0,W_{m}-1) \end{array}$$ (6)

With the 2-D Markov chain model defined by the backoff transition probabilities, the probabilistic theorem of balance equation is used to derive the probability of each state denoted as *S*_*i*,*k*_. The packet transmission probability τ is solved using *S*_*i*,*k*_ where τ is the total amount of every available state probability within the range of *k* ∈ (1, *W*_*i*−1_), *i* ∈ (0,*m*), which is equivalent to one. Finally, by exploiting the normalization condition, the *S*_*i*,*k*_ is determined and simplified as follows:

$$\begin{array}{c} 1=\sum_{i=0}^{m}\sum_{k=0}^{w_{i}-1}S_{i,k}=\sum_{i=0}^{m}S_{i,0}\frac{W_{i}+1}{2}+P_{f}W_{i}-1 \end{array}$$ (7)

The τ can be determined by substituting the equivalent function of the new contention window *W*_*i*_ into Eq 7. In this paper, we extend the 2-D discrete-time Markov chain into two algorithms, (i) BEB sequence with proposed backoff stage resetting process, and (ii) PRSCA (i.e., proposed LFSR sequence with backoff stage resetting process).

The τ for BEB can be derived by substituting the function *W*_*i*_ = *CW*_*min*_ × 2_*i*_ into Eq 7 as follows:

$$\begin{array}{c} S_{0,0}=\frac{2}{\left[\left(1+p\right)+W+2Wp+\frac{p^{3}}{\left(1-2p\right)}(\frac{\frac{p}{1-p}-\frac{p^{m}}{{\left(1-p\right)}^{m}}}{\frac{p^{2}}{{\left(1-p\right)}^{2}}})+\frac{4Wp^{3}}{1-3p}(\frac{\frac{2p}{1-p}-\frac{{\left(2p\right)}^{m}}{{\left(1-p\right)}^{m}}}{\frac{{\left(2p\right)}^{2}}{{\left(1-p\right)}^{2}}})\right]} \end{array}$$ (8)

Then the packet transmission probability is equal to $\frac{S_{0,0}}{1-p}$ and τ can be expressed with the substitution of Eq 8 as follows:

$$\begin{array}{c} \begin{array}{cc} & \tau =\\ & \frac{2}{\left(1-p\right)[\left(1+p\right)+W+2Wp+\frac{p^{3}}{\left(1-2p\right)}(\frac{\frac{p}{1-p}-\frac{p^{m}}{{\left(1-p\right)}^{m}}}{\frac{p^{2}}{{\left(1-p\right)}^{2}}})+\frac{4Wp^{3}}{1-3p}(\frac{\frac{2p}{1-p}-\frac{{\left(2p\right)}^{m}}{{\left(1-p\right)}^{m}}}{\frac{{\left(4p\right)}^{2}}{{\left(1-p\right)}^{2}}})]} \end{array} \end{array}$$ (9)

It is worthwhile to mention that the packet transmission probability for a station in a randomly selected time slot derived in Eq 9 is not similar to the one by Bianchi [7]. This difference lies in the exploitation of the probability of freezing and resetting method of stations in the PRSCA backoff algorithm [6]. For more clarifications, the equivalent substitutions of τ in [7] are shown in Eqs 10, 11 and 12 respectively:

$$\begin{array}{c} 1=\frac{S_{0,0}}{2}[W(\sum_{i=0}^{m-1}{\left(2P\right)}^{i}+\frac{{\left(2p\right)}^{m}}{1-p})+\frac{1}{1-p}] \end{array}$$ (10)

$$\begin{array}{c} S_{0,0}=\frac{2(1-2p)(1-p)}{\left(1-2p\right)\left(W+1\right)+pW(1-{\left(2p\right)}^{m})} \end{array}$$ (11)

$$\begin{array}{c} \tau =\sum_{i=0}^{m}S_{i,0}=\frac{S_{0,0}}{1-p}=\frac{2(1-2p)}{\left(1-2p\right)\left(W+1\right)+pW(1-{\left(2p\right)}^{m})} \end{array}$$ (12)

Interestingly, Bianchi [7] highlighted that when *m* is equal to zero, there will be no consideration of exponential backoff, therefore τ begins to be independent of probability *p*.

Deriving τ for the proposed PRSCA is possible by substituting $W_{i}=2^{I_{i}}\times C_{i}\times CW_{min}$ into Eq 7, in which *C*_*i*_ is the LFSR consecutive numbers formulated in Eq 3, and *I*_*i*_ is the root of double integers formulated in Eq 4. The new packet transmission probability is expressed as follows:

$$\begin{array}{c} \begin{array}{cc} & \tau =\frac{2}{\left(1-p\right)}\times \\ & \frac{1}{\left[\left(1+p\right)+W\left(1+2^{I_{i}}C_{i}p\right)+\frac{p^{3}}{\left(1-2p\right)}(\frac{\frac{p}{1-p}-\frac{p^{m}}{{\left(1-p\right)}^{m}}}{\frac{p^{2}}{{\left(1-p\right)}^{2}}})+\frac{{\left(2^{I}iC_{i}\right)}^{2}Wp^{3}}{1-p(2^{I}iC_{i}+1)}(\frac{\frac{2^{I_{i}}C_{i}p}{1-p}-\frac{{\left(2^{I_{i}}C_{i}p\right)}^{m}}{{\left(1-p\right)}^{m}}}{\frac{{\left(2^{I}iC_{i}p\right)}^{2}}{{\left(1-p\right)}^{2}}})\right]} \end{array} \end{array}$$ (13)

#### 3.4.2 Collision probability and saturated throughput

In principle, τ relies on the probability *p* of conditional and tentative collision. It can be observed that the probability *p* in which a data packet transmission confronts a collision is the probability where in a randomly selected slot of time, at least one of the remaining stations (*n* − 1) is also transmitting [7]. This essential assumption means that every station looks at the network system in a similar state, which is the steady-state of the system. Thus, with the probability of any waiting station can transmit its data packet, τ is as follows:

$$\begin{array}{c} p=1-{\left(1-\tau \right)}^{n-1} \end{array}$$ (14)

From Eq 14, we may consider several events that may occur in a shared medium. The first case is the probability where no station is using the shared medium, or an idle status of the channel can be determined with *P*_*idle*_ [6]. The second case is the indication of at least a single station within a randomly selected slot of time transmits a data packet can be determined through the probability of *P*_*tr*_ [7, 42, 43]. The third case is the probability that a packet is successfully transmitted is determined with *P*_*s*_ [7, 43]. Final case is that multiple stations are attempting to transmit their packets then collision happens, so the probability of a station that collide with other stations (collision probability) can be determined with *P*_*col*_ [43]. All possible probabilities for a predefined number of stations denoted by n are specified respectively:

$$\begin{array}{c} P_{idle}={\left(1-\tau \right)}^{n} \end{array}$$ (15)

$$\begin{array}{c} P_{tr}=1-{\left(1-\tau \right)}^{n} \end{array}$$ (16)

$$\begin{array}{c} P_{s}=\frac{n{\left(1-\tau \right)}^{n-1}}{P_{tr}} \end{array}$$ (17)

$$\begin{array}{c} P_{col}=P_{tr}\left(1-P_{s}\right) \end{array}$$ (18)

Now it is essential to define the saturation throughput ratio *S*_*T*_, as the average payload size of data-bits *E*[*P*] in a given slot duration transmitted successfully. Note that a successful data packet transmission happens in a given time slot with the probability of *P*_*tr*_ and *P*_*s*_. Accordingly, Bianchi [7] expressed the *S*_*T*_ as follows:

$$\begin{array}{c} S_{T}=\frac{E[payload\;bits\;transmitted\;in\;a\;time\;slot]}{\left[length\;of\;the\;time\;slot\right]} \end{array}$$ (19)

The average length of a time slot can be obtained from the expressed probabilities. With the probability of 1 − *P*_*tr*_ the time slot is considered to be unused; while the time slot comprises a successful data packet transmission with the probability of *P*_*tr*_ and *P*_*s*_, and it comprises a data packet collision with the probability of *P*_*tr*_ (1 − *P*_*s*_). Accordingly, the *S*_*T*_ becomes as follows:

$$\begin{array}{c} S_{T}=\frac{P_{s}P_{tr}E\left[P\right]}{\left(1-P_{tr}\right)\sigma +P_{tr}P_{s}T_{s}+P_{tr}\left(1-P_{s}\right)T_{col}} \end{array}$$ (20)

Considering the implementation of a channel access method such as DCF, particularly two equivalent parameters are required to be defined for saturation throughput computation, as shown in the Eq 20, which they are specified as *T*_*s*_ and *T*_*col*_. Firstly, *T*_*s*_ is the time duration where the medium is sensed to be used and busy due to a successful data packet transmission. Secondly, *T*_*col*_ is the time duration where the medium is sensed to be occupied by every station during a data packet collision. However, when a slot is empty, the duration is referred to as *σ*, and certainly the value of each parameter including *E*[*P*] must be formulated with a similar unit. Let *H* = *Phy*_*header*_ + *MAC*_*header*_ be the duration of data packet header, and *δ* be the propagation delay. Both *T*_*s*_ and *T*_*col*_ are given as:

$$\begin{array}{c} T_{s}=H+E\left[P\right]+SIFS+\delta +ACK+DIFS+\delta \end{array}$$ (21)

$$\begin{array}{c} T_{col}=H+E\left[P\right]+DIFS+\delta \end{array}$$ (22)

## 4 Results and discussion

### 4.1 Simulation setup

The performance of the proposed PRSCA backoff algorithm is evaluated and analysed using MATLAB. The simulation uses PRSCA in the DCF access method for calculating new contention window size, with a fixed number of contending stations inside a single RAW slot. The MATLAB simulation consists of the physical layer and the medium access control layer of 802.11ah. The proposed PRSCA is then validated against the work by Bianchi [7] for both collision probability, saturation throughput, throughput, and delay. Moreover, we also implemented the traditional BEB sequences into our backoff stage resetting process (instead of using the LFSR sequence) to analyze the impact of sequences on overall backoff performance. The simulation details can be further elaborated as follows:

1. BEB algorithm is compared with the proposed PRSCA in a WLAN network of 802.11ah with *n* = 50 contending stations, and a non-variable pre-defined payload size of 1 Kbytes (8184 bits).
2. Stations are in a random topology with non-mobility positions in the network.
3. throughput is measured as the amount of successfully transmitted data packets to the time required from one station to another over a communication channel. It is measured in bits per second (bps).
4. delay is measured as the time elapsed between the generation of a frame and its successful reception given by:
  $$\begin{array}{c} D=\frac{n}{S/E[P]} \end{array}$$ (23)
  where *n* is the number of contending stations, *S* is the saturation throughput measured over the average rate of packet payload size *E*[*P*].
5. The 802.11ah standard has defined a new format of frame aggregation in the MAC Service Data Unit referred to as A-MSDU [5], where many sub-frames are aggregated into A-MSDU frames. Therefore, frame aggregation is considered in our simulation.
6. The minimum contention window *CW*_*min*_ for the backoff algorithm is the first input, and the maximum backoff stage *m* is required as the second input.
7. The overall computation parameters of the MATLAB simulation and of Eqs 21 and 22 are summarized in Table 1.

**Table 1:** The MATLAB simulation parameters.

Parameters	Value
*CW*_*min*_	15
*CW*_*max*_	1023
Slot time (*σ*)	52 *μ*s
SIFS	160 *μ*s
DIFS	SIFS + 2 × Slot time(*μ*s)
Station distribution	Random
Number of stations	50
Payload size	1 Kbytes
PHY header	128 bits
MAC header	112 bits
ACK	112 bits
Propagation delay (*δ*)	6 *μ*s
Symbol duration time (Short GI)	36 *μ*s
Symbol duration time (Long GI)	40 *μ*s
Frame aggregation	Enabled
PPDU max time	27840 *μ*s
PSDU max length	797160 bytes
Number of RAW groups	1
Number of RAW slots	1
RAW group duration	0.1 s
Bandwidth	2 MHz
MCS (low to medium data rate)	MCS0 and MCS2
MCS (high data rate)	MCS6 and MCS8

The parameter of m is an indicator of the maximum backoff stage (BOS) used in the simulation. In the BEB algorithm, the largest CW size (*CW*_*max*_) is fixed at 1024. Once BOS arrives at this value, it stops from increasing in the following data packet transmission attempt. For example, the backoff contention window is indicated by *W*_*i*_ = 2^*i*^ × *CW*_*min*_, where *i* takes the values (0, 1, 2, …, m), and *CW*_*min*_ is the minimum CW size which is fixed at a value of 16. Therefore, at BOS of seven where m = 6, the *CW*_*max*_ is measured to be *W*_6_ = 64 × 16 = 1024. We are showing practically the effect of different BOS within our achievable QoS performance parameters measured at different choices of backoff stages for studied algorithms, where maximum BOS can be dealt with as a standardised engineering parameter. The simulation also considered different backoff stages and Modulation and Coding Schemes (MCS) for 2 MHz bandwidth, as shown in Table 2, with randomly distributed stations without mobility in a network environment that is similar to an industrial wireless sensor network in factory automation.

**Table 2:** Data rates of different MCS for 2 MHz channel using 8 *μ*s guard interval.

MCS	Modulation	Code Rate	Data bits per symbol	Data rate (Mbps)
MCS 0	BPSK	1/2	26	0.65
MCS 1	QPSK	1/2	52	1.30
MCS 2	QPSK	3/4	78	1.95
MCS 3	16QAM	1/2	104	2.60
MCS 4	16QAM	3/4	156	3.90
MCS 5	64QAM	2/3	208	5.20
MCS 6	64QAM	3/4	234	5.85
MCS 7	64QAM	5/6	260	6.50
MCS 8	256QAM	3/4	312	7.80

### 4.2 Simulation results and discussion

For extensive analysis, the following backoff algorithms/models were considered:

1. BEB of Bianchi: this model incorporated conventional BEB sequence (i.e., CW is doubled upon every collision until *CW*_*max*_) and traditional resetting process (i.e., to initial window size *CW*_*min*_),
2. BEB of Proposed Model: this model incorporated conventional BEB sequence and the proposed backoff stage resetting process,
3. Proposed PRSCA: this is our proposed PRSCA model, which incorporated the proposed LFSR sequence and backoff stage resetting process.
4. EIED: exponential increase exponential decrease. This model doubles the CW after every unsuccessful transmission, and the CW is halved only after several consecutive successful transmission [44].
5. DIDD: double increment double decrement. This model doubles the CW during a collision and halves the CW during success packet transmission with unlimited number of retransmission attempts [45].

Their performance was evaluated according to four performance parameters, namely the probability of packet collision, saturation throughput, throughput, and average access delay.

#### 4.2.1 Probability of packet collision analysis

**Fig 11:**
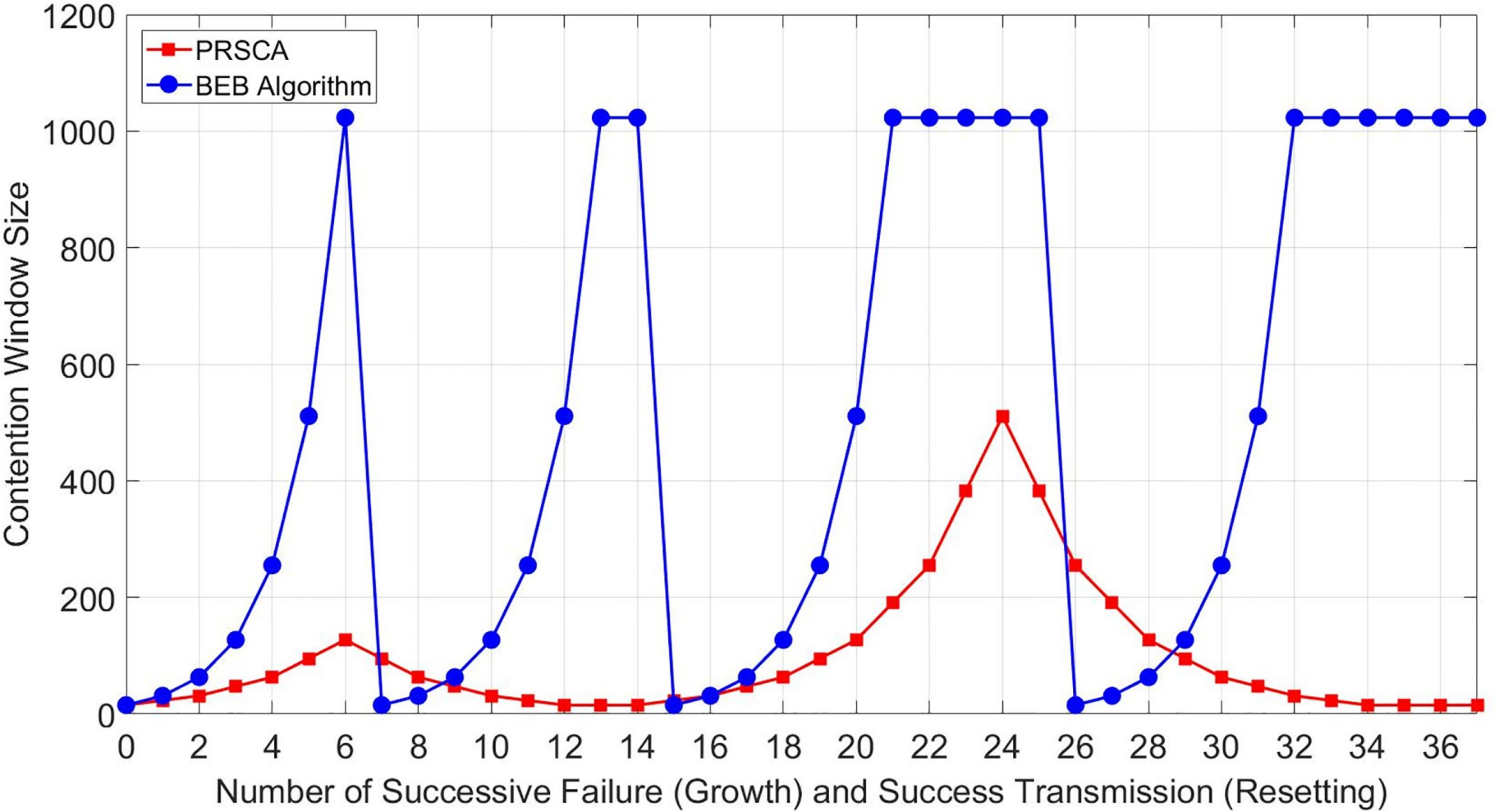
CW growth and reset against number of successive failure and succesful transmission.

To signify the growth and resetting of CWs using the proposed PRSCA algorithm compared to the BEB algorithm, the changes of CW size in a network filled with 400 stations are analysed. Fig 11 shows the CW size after successive collision/contention failure and eventual successful transmission. Station with successful transmission using BEB will reset its CW to *CW*_*min*_, while station using the PRSCA algorithm only reduce its backoff stages by 1. Therefore, BEB shows very drastic changes in the CW size compared to PRSCA, from absolute maximum to minimum CW size. Furthermore, the sequence used by PRSCA also warrants the gradual increase of CW size, whereby it reduces the time wasted for medium sensing.

**Fig 12:**
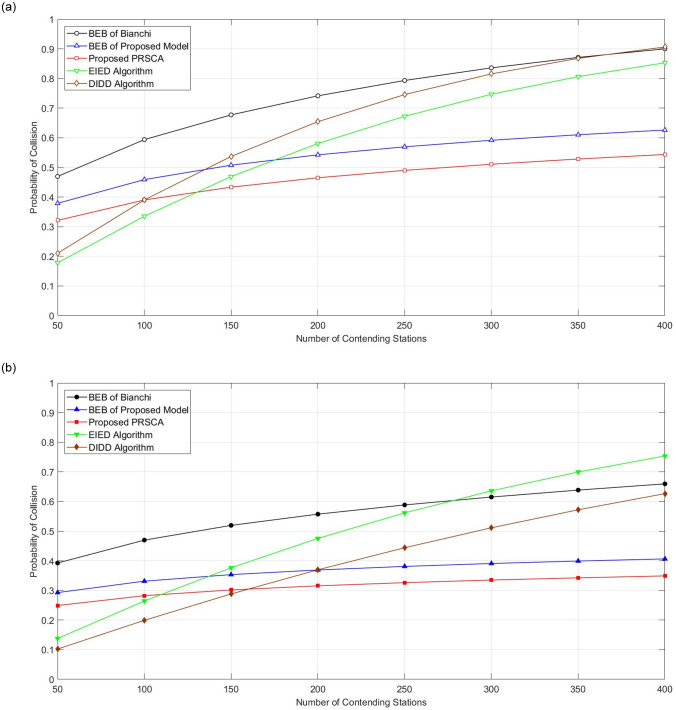
Collision probability. (a) fifth BOS, where m = 4; (b) seventh BOS, where m = 6.

Fig 12 illustrates the collision probability of two backoff stages, namely BOS of five (Fig 12a) and seven (Fig 12b). Note that the numbers (i.e., five and seven) indicated the maximum allowed backoff stages. From Fig 12, it is apparent that the proposed PRSCA algorithm achieves lower collision probability for all number of stations compared to BEB. In particular, at fifth BOS, the improvement is approximately 15.5% compared to BEB of Bianchi and 6% compared to BEB of the same model; and at seventh BOS is offers 18% and 4.5%, respectively compared to BEB of both models. The amount of reduction in the collision probability is clearer in the higher backoff stages because of the higher frequency of the BEB algorithm using the resetting mechanism upon higher collisions probability. But PRSCA is using a different mechanism of resetting, which is instead of going back to the initial stage, the station steps back to the prior stage, whereas a station is able to effectively reduce the probability of collisions. However, for the network with lower density (< 150), EIED and DIDD are outperforming the proposed PRSCA model. This is mainly due to the different sequence employed by the algorithm. To avoid a drastic increase of the BEB sequence and the subsequent delay caused, PRSCA is using the LFSR sequence for CW size. The slower growth rate of the LFSR sequence compared to the BEB sequence used by EIED and DIDD caused the network to experience a higher collision. However, as the network size increased, BOS of the station using PRSCA will be maintained at a higher value. Therefore, a higher value of PRSCA is used that helps reduce the probability of collision.

A major cause of network performance degradation is due to packet collisions, which is defined as the probability of a packet that encounters a collision with another packet due to simultaneous transmission. Therefore, any increasing or decreasing in the probability of collided packets will decide the probability of unsuccessful packet transmissions. In the BEB algorithm, whenever a collision happens in the medium, the contention window (CW) size increases in an exponential manner. In a way that forces the station to sense the medium for a longer duration in order to start its data packet transmission. Meanwhile, other stations with a newly arrived packet for transmission and a lower size of CW simultaneously sensing the medium to get the channel access. Unaware of the collisions experienced by stations under the same coverage area, the lower CW size of these stations caused them to experience collision due to the saturated network condition. Consequently, in using the BEB algorithm, the stations engaged in the process of doubling the CW size, which leads to degradation in network performance in terms of energy and time.

#### 4.2.2 Saturation throughput analysis

In order to analyze the impact of the proposed PRSCA on saturation throughput, we fixed the number of contending stations and assumed saturated conditions appeared at every station. In particular, the stations always have data frame that is pending for transmission. Moreover, we assumed there is no hidden stations and noise to corrupt the data frame, which is waiting for transmission. Thus, any data frame transmission failure is only due to the collision that happens when stations are transmitting at the same time.

**Fig 13:**
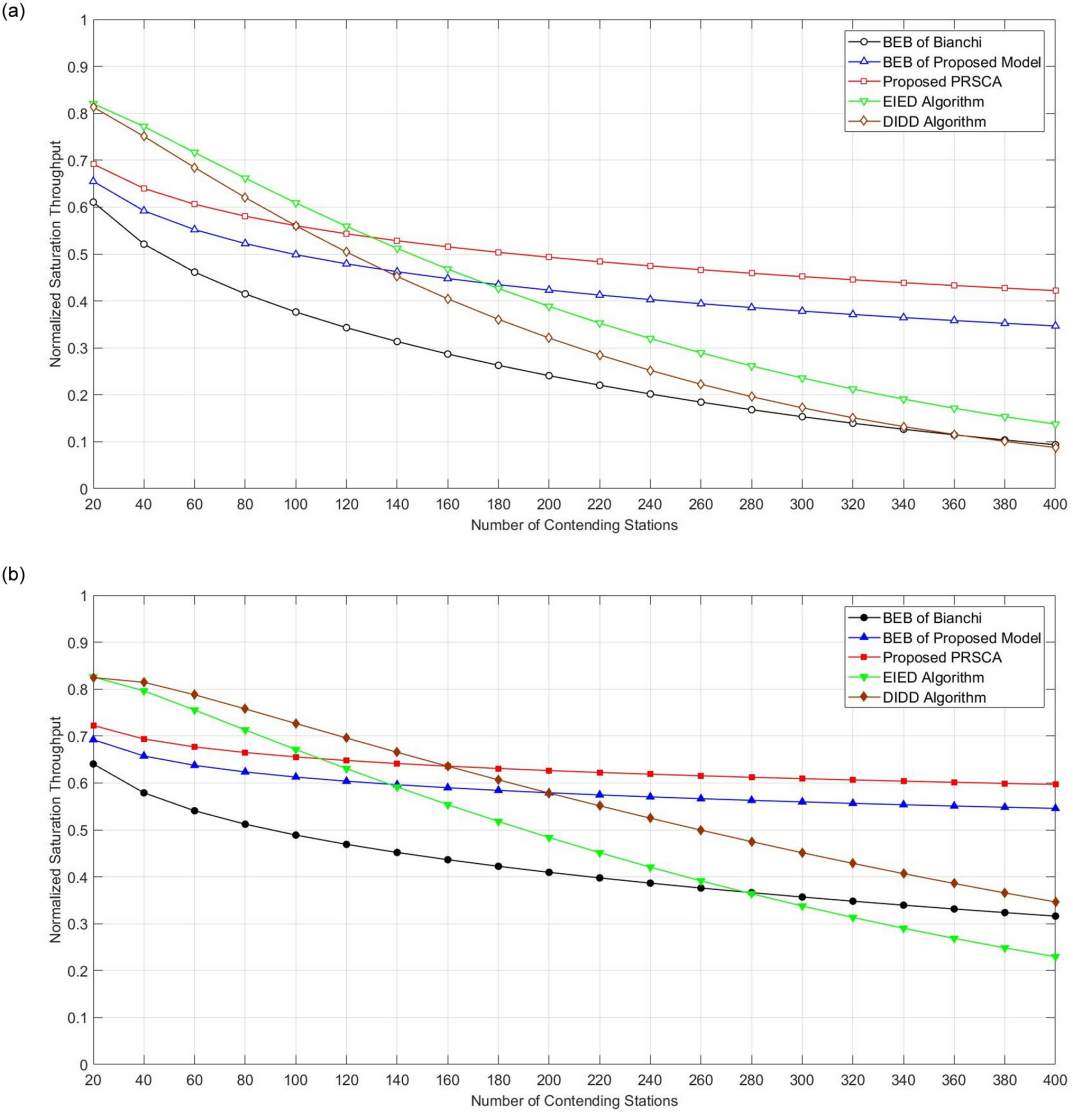
Normalized saturation throughput. (a) fifth BOS, where m = 4; (b) seventh BOS, where m = 6.

Fig 13 illustrates the normalized saturation throughput for the proposed PRSCA algorithm, conventional BEB, BEB within PRSCA, EIED, and DIDD; with BOS of five and seven. From the figure, it is apparent that the proposed PRSCA with the model is offering the best performance compared to the other algorithms. The result also shows that the saturation throughput is decreasing when there is an increase in the number of contending stations. From our further analysis of the simulation, we found that this is due to the higher number of collisions that happened at a higher number of stations.

It can be observed that the normalized saturation throughput is around 0.68 and 0.72 for the proposed PRSCA at BOS of five and seven respectively, when 25 stations are competing under the DCF access mechanism. Based on this result, we found that the PRSCA obtained an improvement rate of 13.5% in the saturation throughput performance compared to Bianchi’s model, and higher than BEB of PSRCA model by 5.5% at BOS of five. The same pattern occurred to BOS of seven. These improvements are due to having a lower number of data packet collisions from the removal of resetting mechanism to initial BOS and the CW update with every collision using PRSCA. It is also clear that the impact of the number of stations on the proposed PRSCA was relatively less significant compared to the BEB of both models. In other words, PRSCA is performing much better at higher backoff stages.

Compared to EIED and DIDD, the observation from Fig 13 is similar to those from the probability of packet collision in Fig 12. In the network with lower density (< 160), EIED and DIDD have better saturation throughput. In a network filled with 20 stations, the improvement of saturation throughput can reach up to approximately 13%. However, the advantage of PRSCA starts to become more apparent as the number of station increase. This is due to the effectiveness of the LSFR sequence employed in the proposed algorithm. When the number of stations is 400, the improvement of PRSCA over EIED and DIDD is at staggering 29% and 39% respectively at seventh BOS. This observation demonstrated the effectiveness of PRSCA for channel access in future dense wireless networks.

It is important to highlight that, in a dense network, contending stations are more likely to contend for channel access successfully with high CW. To reduce the time wasted on lower BOS, PRSCA has enhanced the backoff resetting process by avoiding the reset of BOS to initial value. This prevention allows the CW size to be preserved at the value relevant to the current network condition. While larger CW size means the station will spend more time on medium sensing or more delay, the avoidance of collision has outweighed this delay. This situation is proven by the improvement of saturation throughput shown in Fig 13.

#### 4.2.3 Throughput analysis

In this section, we analyzed the impact of the proposed algorithm on the throughput of the 802.11ah network. In particular, we focused on two cases according to their data rate, where MCS0 and MCS2 represented low and medium data rate cases respectively, and MCS6 and MCS8 represented high data rate cases. In industrial IoT scenarios such as factory automation application, AP of the network is required to meet the data traffic requirements of various small and low-cost sensors [46]. These sensors respond to the physical condition and produce measurements such as current, voltage, fuel, pressure, temperature, humidity, levels of CO2, oil readings, and so on. In the case of low data rate transmission, they are used for handling non-realtime data where throughput is relatively not critical.

**Fig 14:**
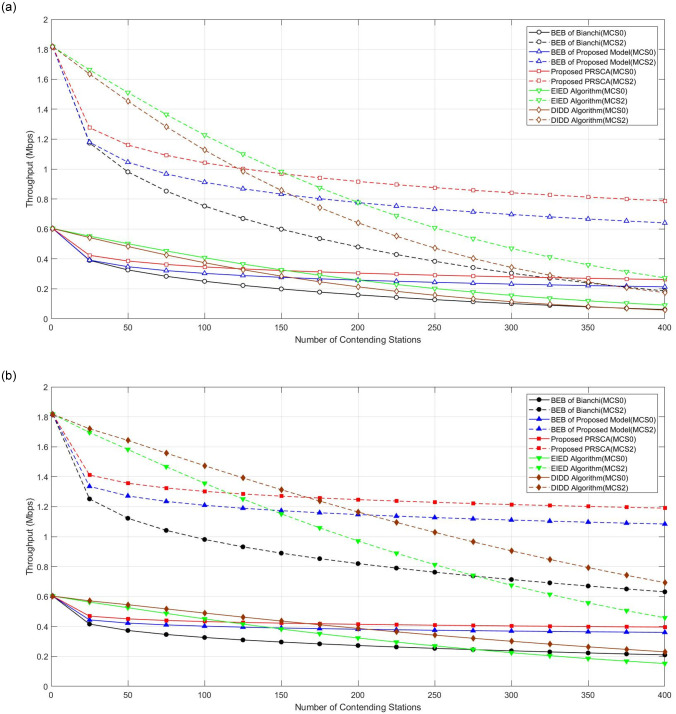
Throughput for different algorithms considering MCS0/MCS2. (a) At fifth BOS, where *m* = 4, indicated by hollowed markers with solid and dashed curves, respectively; (b) At seventh BOS, where *m* = 6, indicated by filled markers with solid and dashed curves, respectively.

The throughput performance for studied algorithms at lower MCS for different backoff stages is depicted in Fig 14. In general, the throughput performance degraded when the number of contending stations increases. However, from this figure, it can be observed that the PRSCA offers better throughput performance compared to the BEB algorithm of the same model and Bianchi’s model. For example, at BOS of seven (refer to Fig 14b), improvement of the throughput at MCS2 is 5% for the proposed PRSCA, whereas 3% for BEB algorithm of Bianchi’s model. Nonetheless, the shortcomings of PRSCA compared to EIED and DIDD in lower density network (< 150) is observed, similar to the observation in packet collision and saturation throughput. In a high-density wireless network, PRSCA is undoubtedly the algorithm with the best performance.

**Fig 15:**
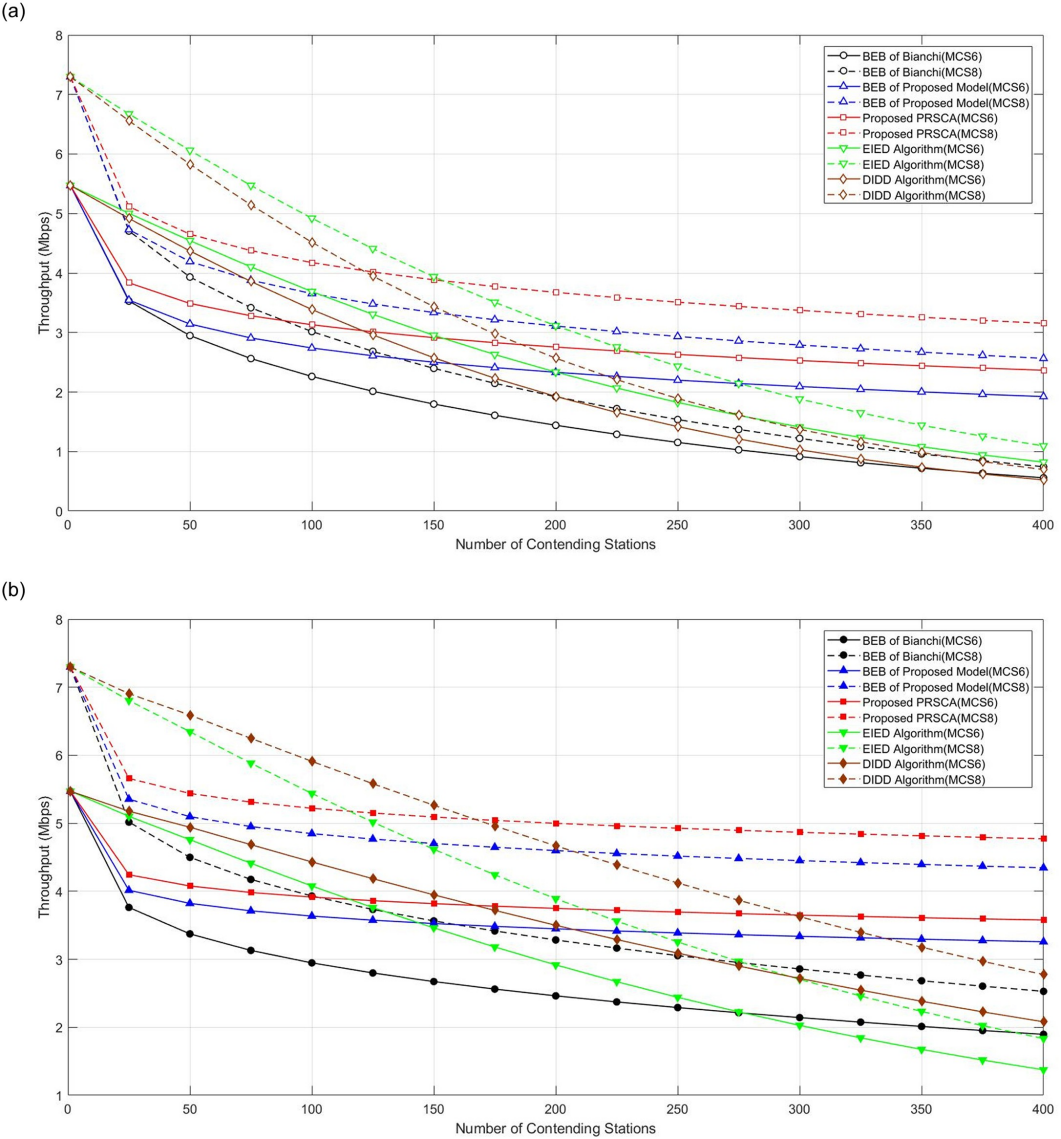
Throughput for different algorithms considering MCS6/MCS8. (a) At fifth BOS, where *m* = 4, indicated by hollowed markers with solid and dashed curves, respectively; (b) At seventh BOS, where *m* = 6, indicated by filled markers with solid and dashed curves, respectively.

Next, the effectiveness of the proposed algorithm has been analysed and evaluated for higher data rates. High data rate transmission normally deals with complex applications in factory automation, which combines high-speed communication with real-time control [47]. In addition, they are normally used for time-sensitive data information. Fig 15 illustrates the impact of PRSCA and BEB algorithms on throughput at higher MCS (in this case, MCS 6 and MCS8). From the figure, it is apparent that the PRSCA again outperformed the BEB algorithm of both models. Similar to throughput for different algorithms with lower data rates, PRSCA outperforms EIED and DIDD in a denser wireless network, whilst EIED and DIDD perform better in the network with a lower number of stations.

#### 4.2.4 Delay analysis

**Fig 16:**
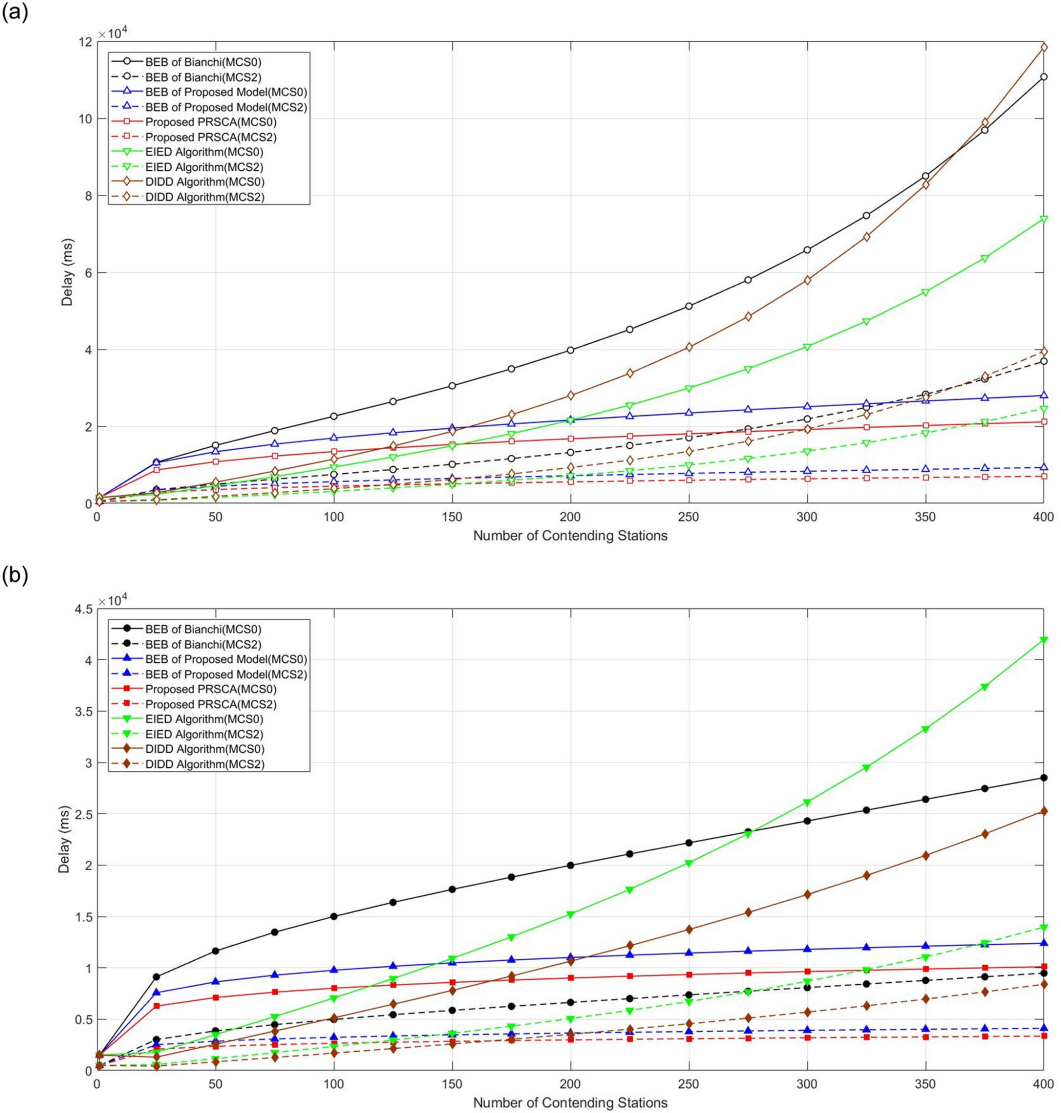
Average access delay. (a) At fifth BOS, where *m* = 4, indicated by hollowed markers with solid and dashed curves, respectively; (b) At seventh BOS, where *m* = 6, indicated by filled markers with solid and dashed curves, respectively.

This section highlights the delay performance of the proposed algorithm for low (MCS0 and MCS2) and high data rate (MCS6 and MCS8) transmission. The simulated average access delay of studied algorithms for low MCS is depicted in Fig 16. Obviously, the higher the number of contending stations, the longer the delay. In this case, sensor-based stations that are not time-sensitive won’t get affected by this high amount of delay. Sometimes, this kind of station is also categorised as the best effort station.

Given that the delay is depending on the time needed to complete the packet transmission, higher data rates promises lower delay. Therefore, regardless of the algorithm used, MCS8 shows the lowest expected delay. From Fig 16, PRSCA again shows the best performance, especially in a dense wireless network (*n* = 400). It is interesting to note that DIDD and EIED are among the worst performers in a dense wireless network (*n* = 400). This is due to the inflexibility of their backoff stage resetting process, especially EIED. In EIED, the CW size will only be halved after several consecutive successful transmissions. In a dense wireless network, collision and unsuccessful transmission are more likely to occur, hence, reduce the chance of CW size being reduced.

**Fig 17:**
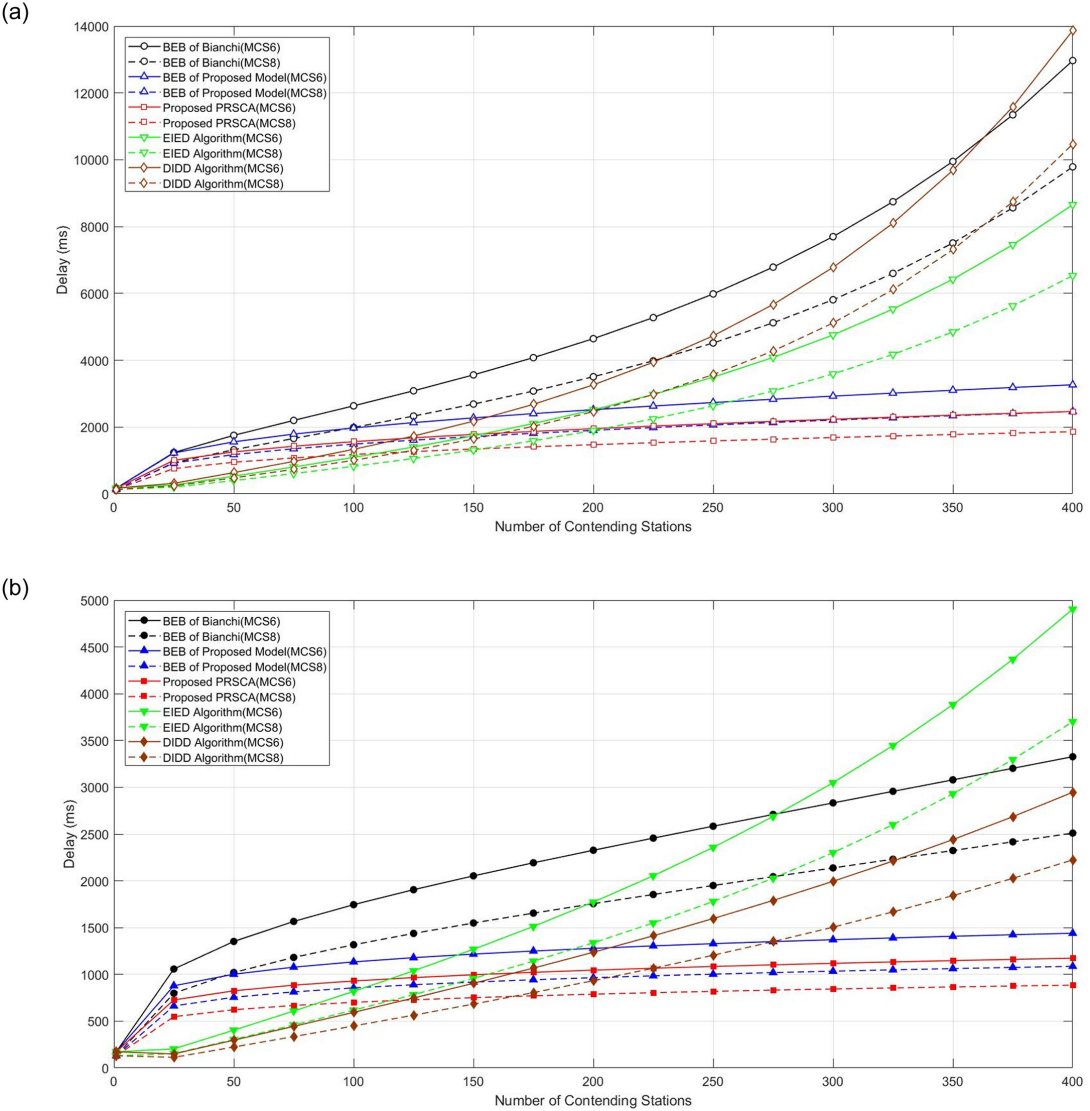
Average access delay. (a) At fifth BOS, where *m* = 4, indicated by hollowed markers with solid and dashed curves, respectively; (b) At seventh BOS, where *m* = 6, indicated by filled markers with solid and dashed curves, respectively.

Meanwhile, Fig 17 illustrates the average delay performance for PRSCA and the other models for high data rates, namely MCS6 and MCS8. Similar to the previous low data rate, we can observe that the same pattern applies in this simulation. It is apparent that the average delay increases as the number of contending stations increases. Moreover, with respect to delay at higher MCS, the proposed PRSCA offers better performance compared to other studied algorithms.

Interestingly, EIED and DIDD models show better results at a lower number of contending stations (i.e., below 150). However, when the number increases, the proposed algorithm achieved better results and showed stable performance with around 2 seconds and 1 second of delay for BOS at 5 and 7 respectively. As explained earlier, in the BEB model, after every successful transmission, the station goes back to the initial stage in which leads to more collision. This issue is mitigated by allowing stations to move from present BOS to the prior BOS using the proposed model. Moreover, the proposed algorithm uses a pseudorandom sequence that has a slow increment when it calculates CW size rather than increasing exponentially by doubling it whenever a collision happens. The probability of freezing is also adopted whenever a station senses a busy medium.

## 5 Conclusions

This paper introduced PRSCA, an efficient algorithm to be used within the RAW slot (or contention-based component) of the IEEE802.11ah standard by focusing mainly on providing better contention resolution and resetting mechanism to contending stations in the network. Unlike the conventional approach where the CW is doubled upon every collision, the proposed PRSCA utilized the LFSR sequence, which suits congested networks as it provides a suitable rate of growth in CW and eventually reducing the probability of collisions. We modelled the proposed PRSCA using a discrete 2-D Markov chain, inspired by the well-known Bianchi’s model [7]. In addition, an essential modification has been carried out on the conventional resetting process. In PRSCA, to reduce collisions and degradation of network performance, we adjusted the resetting process where a station moves to the prior backoff stage instead of returning to the initial backoff stage. The performance and robustness of PRSCA were evaluated by considering various scenarios; the number of contending stations, modulation and coding schemes (MCS), and backoff stages. To better demonstrate the superior performance of the proposed PRSCA, and validate our motivations and arguments, it is compared with two other backoff mechanisms regarding several fundamental QoS metrics such as the probability of packet collision, saturation throughput, throughput, and delay. From the results, it is evident that the proposed PRSCA offered the best overall performance compared to the other two backoff mechanisms through a more developed solution, while in practice, it can be implemented easily. Our analysis of the proposed PRSCA also yields interesting observations on the significant impact of the backoff sequence and resetting mechanism on the overall performance of the network.

## Supporting information

S1 Dataset Minimal dataset. Data required to replicate all study findings reported in the article.
(XLSX)
